# Ligand Basicity Governs Cysteine Reactivity in Au(I)–NHC Thiolate Complexes: A Computational Study

**DOI:** 10.1021/acs.inorgchem.5c02777

**Published:** 2025-09-26

**Authors:** Gustavo Clauss, Igor Santos Oliveira, Camilla Abbehausen

**Affiliations:** Institute of Chemistry, State University of Campinas, Campinas 13083-632, Brazil

## Abstract

Gold–thiolate compounds have emerged as promising therapeutic agents, showing activity against parasites such as *Leishmania amazonensis* and *Trypanosoma cruzi*, as well as viruses including Mayaro, Zika, and SARS-CoV. However, their use is limited by speciation due to the rapid ligand exchange with thiolated biomolecules, and control of this reactivity is key for the design of drugs. This study explores the reactivity of linear Au­(I) complexes featuring the NHC, 1,3-bis­(mesityl)­imidazole-2-ylidene (IMes), and thiol-donating ligands, specifically pyrimidine-2-thione (HSpym), 2-thiouracil (2tuH), 1,3-thiazolidine-2-thione (HStzn), and 1,3-benzothiazole-2-thione (HSbtz), along with a chloride complex, Au­(IMes)­Cl, focusing on their interaction with the biologically significant amino acid cysteine. Thiol-donor ligands were selected for their structural diversity, biological relevance, and presence in drug-like molecules, allowing a systematic comparison of their reactivity with cysteine. Using DFT, we calculated Gibbs free energy variations that corresponded well with experimental equilibrium data from the ^1^H NMR measurements. Electronic structural parameters and ligand basicity provided insights into the observed thermodynamic trends, with complexes containing the most basic ligands, Stzn and 2tu, reacting more extensively with cysteine. Among three proposed reaction mechanisms, we identified a preferred pathway, suggesting that the leaving ligand may participate in cysteine deprotonation. In this mechanism, the initial exchange with protonated cysteine was most favorable for Au­(IMes)­Spym, while deprotonation of the intermediate proceeded most rapidly with Au­(IMes)­Cl. These findings elucidate the influence of ligand characteristics on heteroleptic Au­(I)­(NHC) complex reactivity, contributing to the understanding of these complexes as prospective therapeutic agents.

## Graphical abstract

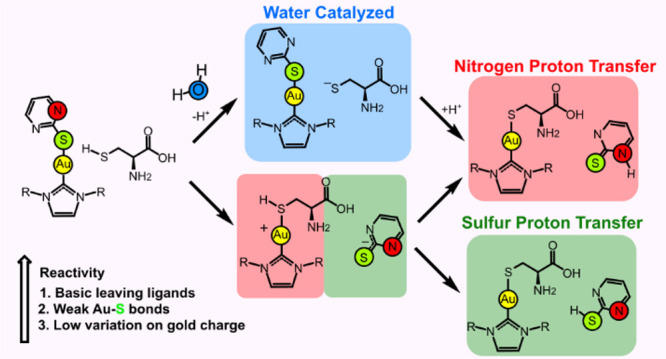

## Introduction

Gold-based drugs have a long history in medicinal applications, culminating in the FDA approval of gold-based compounds for the treatment of rheumatoid arthritis.^1–3^ Thereafter, Au­(I) and Au­(III) complexes demonstrated significant activity against several diseases due to their ability to interact with thiol- and selenol-rich biomolecules,^4,5^ inhibiting several validated targets.^6,7^ However, the gold reactivity leads to speciation and nonspecific binding, complicating their mechanism of action and raising concerns about toxicity and off-target effects.^4,8,9^

In our group, we have developed and studied Au­(I)­(NHC) complexes, with varied ligands, as antivirals, antiparasitic, and zinc finger inhibitors.^7,9–11^ We have found significant antiviral and antiparasitic activity in a series of Au­(I)­(NHC)Cl and Cu­(I)­(NHC)­Cl. We noted that the biological activity of these complexes is intrinsically connected to their reactivity in ligand exchange processes.^10,12^

Looking at the ligand exchange of Au­(I)­(NHC)Cl with *N*-acetylcysteine (NAC), we found generally low reactivity, which appears to be linked to low toxicity observed for host cells.^10^ In recent work, we explored the substitution of the chloride ligand in Au­(I)­(NHC)Cl with thiol-donor thiopyrimidine and thiazolidine ligands. These ligands were selected not only because they are present in several FDA-approved drugs^13^ but also due to their natural occurrence and structural features that have inspired their use in the design of biological probes, and peptidomimetics.^13,14^ Additionally, the choice of these structurally related thiol ligands allows a systematic comparison of the activity of the resulting complexes. This rationale aligns with our aim to introduce ligands capable of modulating reactivity while retaining biological relevance, as exemplified by the thiosugar moiety in auranofin.^15^ We observed that replacing chloride with these thiol ligands facilitated ligand exchange with NAC; however, this also increased toxicity toward host cells, thereby reducing selectivity.

Several studies in the literature have employed density functional theory (DFT) calculations to explore the reactivity of Au­(I) complexes with reduced models of biomolecules, including free amino acid molecules,^16,17^ side-chain groups,^18–20^ and even small peptides.^18,21^ According to Tolbatov and Marrone, the use of single-molecule models can be a valuable tool when the target residue is solvent-exposed on the protein surface.^22^ However, this approach may introduce artifacts in cases where the residue is buried within the enzyme’s active site or when significant steric hindrance is present. This presents a paradox in the context of gold complex reactivity in biological environments: While selective reactivity toward the intended target is essential, the gold complexes must simultaneously remain inert toward abundant thiol-rich biomolecules.

Previously, our group investigated the reaction mechanism of Au­(IMes)­Cl with a cysteine (Cys) molecule through DFT calculations using a simplified model that considered a single-step reaction with deprotonated Cys.^17^ Studies of other Au­(I) complexes place significant emphasis on proton transfer in ligand exchange reactions, considering multistep mechanisms that can deliver more accurate thermodynamic and kinetic values.^16,23–25^ Tolbatov and co-workers demonstrated that ligand exchange in [Au­(NHC)_2_]^+^ complexes requires attack by thiolate or selenothiolate species, with explicit proton transfer to the leaving carbene.^25^ Šebesta and co-workers highlighted the strong pH dependence of the mechanism for Au­(NHC)Cl complexes with thioredoxin reductase models, emphasizing the role of Cys and selenocysteine protonation states.^24^ Nevertheless, the potential role of proton transfer to a labile sulfur-containing leaving ligand has not yet been considered in previous studies, and we have seen that it can affect the biological outcome through exchange with biomolecules.

In this work, we employed DFT and mechanistic multistep pathways to investigate the ligand exchange reactions of a series of Au­(I)­(IMes)­X complexes, where X represents various sulfur-containing ligands, including thiopyrimidines and thiazolidines: thiazolidine-2-thione (HStzn), 1,3-benzothiazole-2-thione (HSbtz), pyrimidine-2-thione (HSpym), 2-thiouracil (2tuH), and chloride (Figure 1).^10^ The exchange reactions were studied in the presence of NAC as the incoming ligand to identify trends explaining the thermodynamics of these reactions. Building on previously reported experimental equilibrium data, this study provides new theoretical insight into how ligand basicity influences both the thermodynamics and the kinetics of ligand exchange. Notably, among the proposed mechanisms, we highlight a new plausible pathway in which the leaving ligand participates in Cys deprotonation, offering a deeper understanding of the factors controlling the Au­(I)­(NHC) complex reactivity.

**Figure 1:**
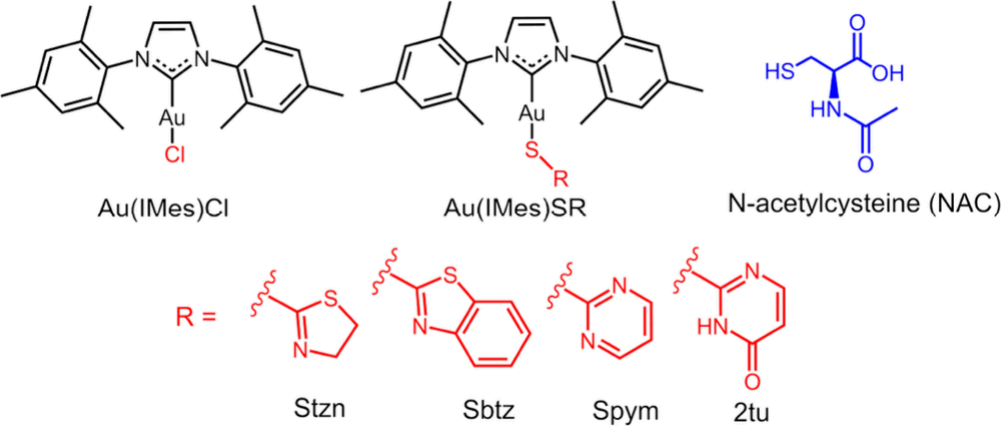
Structures of Au­(IMes)Cl and Au­(IMes)­SR, where SR = Stzn, Sbtz, Spym, and 2tu, and NAC (NAC).

## Computational Details

All DFT calculations were performed using the ORCA package version 6.0.^26–28^ Furthermore, Mayer bond orders and steric volume were calculated for all molecules of interest using Multiwfn software.^29^

### Thermodynamics

All reactants had their structures optimized using DFT, including the gold complexes (Au­(IMes)­Cl, Au­(IMes)­2tu, Au­(IMes)­Sbtz, Au­(IMes)­Spym, and Au­(IMes)­Stzn), cysteine (Cys), and deprotonated cysteine (Cys^–^), leaving ligands in their protonated HX (HCl, 2tuH, SbtzH, SpymH, and StznH) and deprotonated X^–^ (Cl^–^, 2tu, Sbtz, Spym, and Stzn) forms. For 2tuH, SbtzH, SpymH, and StznH, both thioamide and thioiminol tautomers were considered, for further stability comparison. All calculations employed the PBE0 functional,^30^ SARC-ZORA-TZVP basis set for Au­(I) cations,^31^ ZORA-def2-TZVP basis set for other atoms,^32^ SARC/J auxiliary basis set, RIJCOSX approximation,^33^ CPCM for implicit DMSO solvation,^34,35^ and a convergence criterion of 1.0 × 10^–8^ a.u. Frequency calculations were performed on the optimized structures for minima verification and to calculate thermodynamic data, at 298.15 K. The Gibbs free energy variation (Δ*G*) was then calculated based on the difference in free energy of products (Au­(IMes)­(Cys) and HX, the ligand in the protonated form) and reactants (Cys and Au­(IMes)­X, where X = Cl, 2tu, Sbtz, Spym, and Stzn). Experimental Δ*G* were determined from equilibrium constants (*K*) obtained by ^1^H NMR spectroscopy.^10^ The equilibrium between the Au­(I)–NHC–thiolate complexes and NAC was monitored by integrating the CH_3_ signal of free and coordinated NAC in reactants and products, respectively. The *K* values were calculated from the relative signal intensities and converted to Δ*G* using the relation Δ*G* = −*RT* ln *K*, at 298.15 K.

### p*K*_b_ Estimate

The p*K*_b_ values for all leaving ligands were calculated via the direct method.^36^ This was achieved by estimating the energy of the solvated hydrogen ion in DMSO, using experimental p*K*_a_ values from a set of 40 amides and thioamides.^37^ Gibbs energies (G) for HA (the protonated form) and A^–^ (the conjugate base) were obtained through geometry optimization and frequency calculations at the same level of theory used for the Au­(IMes)­X complexes. Here, HA represents general amide or thioamide structures of the form RC­(=Z)­NR′R″, with Z = O or S, and R′, R″ = H, alkyl, or part of a ring. Then, 40 *G*(H^+^_(DMSO)_) values were calculated using eq 1.^38^ *G̅*(H^+^_(DMSO)_) was determined as the average of the energies obtained for the 40 amides. To calculate the leaving ligands p*K*_b_ values, *G*(HX_(DMSO)_) and *G*(X^–^_(DMSO)_) were obtained from optimized ligand HX and X^–^ structures and calculated using eq 2.

$$G\left(H_{\left(\mathrm{DMSO}\right)}^{+}\right)={\left(pK_{a}^{\exp}\right)}^{*}RT\ln\;10+G\left({\mathrm{HA}}_{\left(\mathrm{DMSO}\right)}\right)-G\left(A_{\left(\mathrm{DMSO}\right)}^{-}\right)$$ 1

$$pK_{B}=14-\mathrm{G̅}\left(H_{\left(\mathrm{DMSO}\right)}^{+}\right)-G\left({\mathrm{HX}}_{\left(\mathrm{DMSO}\right)}\right)+G\left(X_{\left(\mathrm{DMSO}\right)}^{-}\right)$$ 2

### Kinetics

For kinetics evaluation, three multistep mechanisms were considered and are represented in Figure 2: one involving water as a catalyst (H_2_O-PT), where a molecule of H_2_O deprotonates Cys to form Cys^–^ before the ligand exchange, and two others where the ligand exchange occurs with Cys, resulting in an intermediate Au­(IMes)­(CysH)^+^ complex, which then has a hydrogen abstracted by the leaving ligand—one via nitrogen proton transfer (NPT) and the other via sulfur proton transfer (SPT). Unlike the other ligands, Cl^–^ is not ambidentate; therefore, the NPT and SPT mechanisms are equivalent.

**Figure 2:**
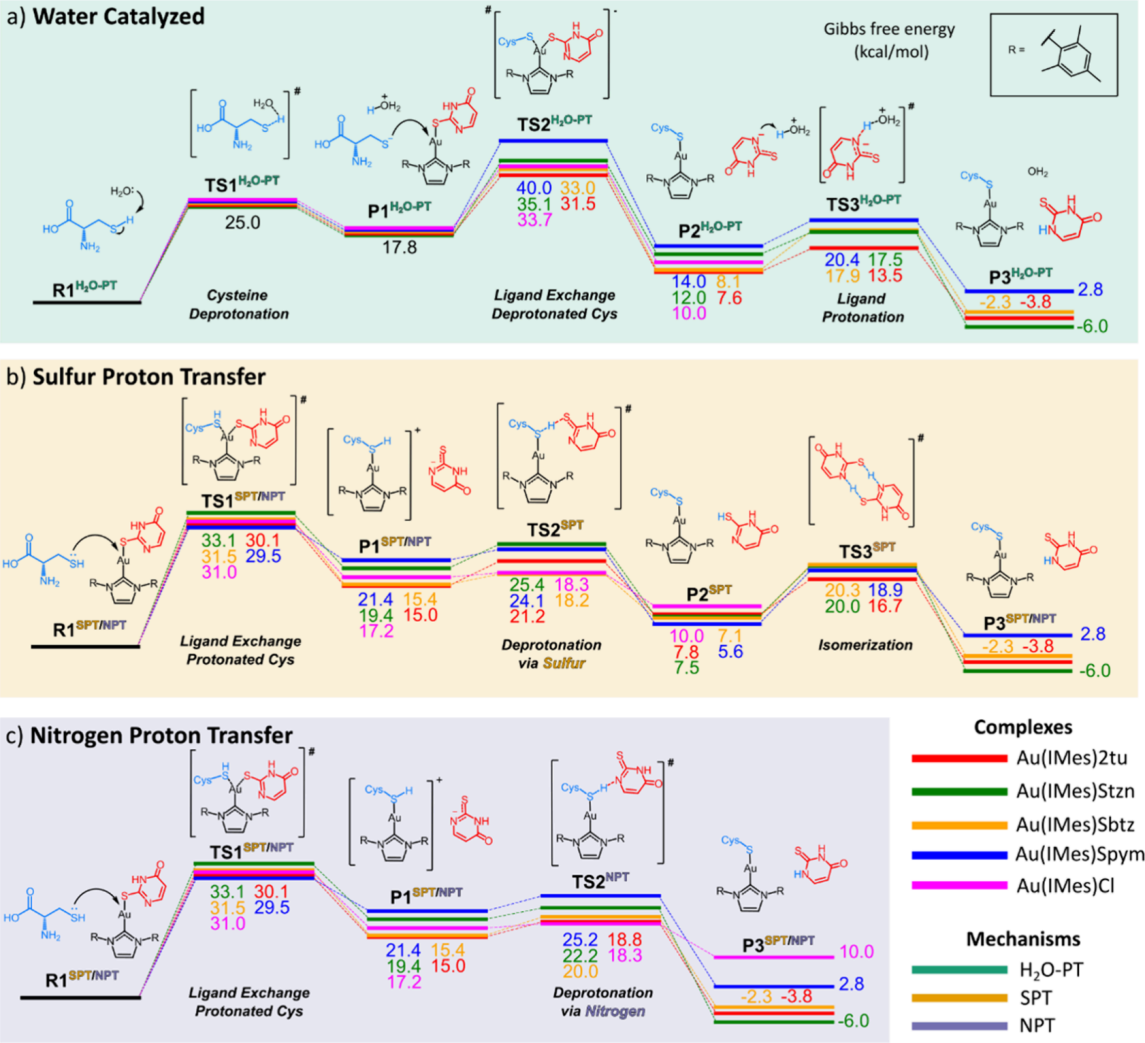
Calculated energy profile for three mechanisms of Au­(IMes)­X reaction with Cys, where X = 2tu, Sbtz, Spym, Stzn, and Cl. (a) SPT, (b) NPT, and (c) H_2_O-PT.

Transition states (TS) were identified using the relaxed surface scan method. The scans were performed in 11 steps using DFT, employing the same parameters as those used for optimizing the reagent and product structures. From the scan results, one to two candidate structures were selected as potential TS and further optimized at the DFT level. This optimization focused on following the lowest energy eigenvalues corresponding to the reaction coordinates of interest by applying the same theoretical levels in all steps. Intrinsic reaction coordinate (IRC) calculations were employed to confirm that the optimized TS correctly connects the reactants and products. With the TS structures confirmed, the Δ*G* of activation for each step was determined by subtracting the Δ*G* of the isolated reactant species from that of the corresponding TS.

## Results and Discussion

### Thermodynamics

Optimizations of the free ligands indicated that the thione isomers are generally more stable than their thioiminol counterparts. However, the energy difference between these isomeric forms varies considerably. The thione form is favorable by 13.5, 11.7, 9.5, and 2.8 kcal/mol for StznH, 2tuH, SbtzH, and SpymH, respectively, as reported in our previous work.^10^ This trend is consistent with findings in the literature, including previous work by our group, which shows that the thione form is preferred in polar solvents such as DMSO.^39,40^

Given that thione forms are more stable than thioiminol forms, variations in the (Δ*G)* of reactions between Au­(IMes)­X and Cys considered the formation of HX in the thione form. Table 1 shows the Δ*G* (in kcal/mol) for the reactions.

**Table 1:** Calculated Δ*G* for the Reaction of Au­(IMes)­X with Cys, and Correlation with Experimental Δ*G*, X HOMO–LUMO Gap, Estimated p*K*_b_, and Au-X Mayer Bond Order^a^

	Δ*G* (kcal/mol)				
complex	experimental	calculated	X HOMO–LUMO gap (eV)	Au–X bond order	estimated p*K*_b_ of X
**Au(IMes)Cl**	2.4	10	17.6	0.812	18.1
**Au(IMes)Spym**	–0.6	2.8	4.8	0.883	5.8
**Au(IMes)Sbtz**	–1.5	–2.3	4.9	0.878	6.5
**Au(IMes)2tu**	–3.9	–3.8	5.1	0.830	5.6
**Au(IMes)Stzn**	–5.5	–6	6.2	0.899	0.8
**Au(IMes)Cys**	N/A	N/A	5.5	0.967	0.9

a pKb was calculated using *G̅*(H^+^_(DMSO)_), (−275 ± 2) kcal/mol (Table S1).

PBE0 was selected because it has been successfully applied in our previous studies involving gold­(I) complexes.^10,17^ In addition, the following functionals were tested: B3LYP, which is widely used and recognized for its performance in organometallic chemistry, and ωB97X-D3BJ and M06-2X, which were employed in a benchmarking analysis on the reaction between gold­(I) and Cys by Tolbatov et al.^25^ These range-separated hybrid functionals are designed to improve the description of long-range exchange interactions and noncovalent effects, which are particularly relevant to metal–ligand bonding and intermolecular interactions. However, it was not possible to identify a single best-performing functional, as all of them yielded similar deviation values for structural parameters^10,41^ (Table S2) and Δ*G* (Table S3) compared to experimental values. The optimized structures of the complexes are represented in Figure S1.

In a previous model, the Δ*G* calculation for the Au­(IMes)Cl and Cys reaction assumed HCl as the product, without accounting for its degree of dissociation.^10^ In this work, we refined the estimation by including the energy of the solvated proton and incorporating the dissociation in the Δ*G* estimation. When HCl was considered as a product, the reaction Δ*G* was 15.2 kcal/mol. However, when the dissociated form H^+^ and Cl^–^ were considered, Δ*G* decreased to 10.0 kcal/mol, indicating a more favorable thermodynamic outcome and providing a more accurate estimation of the reaction energetics.

The calculated and experimental Δ*G* values obtained from the ligand exchange ^1^H NMR assay show good agreement . The largest deviations are observed for Au­(IMes)Cl and Au­(IMes)­Spym. For Au­(IMes)­Cl, Δ*G* is overestimated, likely due to limitations in modeling the solvation energy of Cl^–^ in DMSO using the CPCM model. For Au­(IMes)­Spym, Δ*G* is underestimated, possibly due to parallel equilibria leading to the experimental formation of NAC-coordinated complexes, such as through conversion of the leaving ligand into its thioiminol tautomer. Since Spym has the smallest tautomeric energy gap (2.8 kcal/mol), both equilibria may occur simultaneously.

Although the experimental ^1^H NMR assays were conducted with NAC, the calculations here employed Cys instead. This choice was made because Cys is a smaller molecule, simplifying the calculations. Experiments were conducted with NAC to avoid interaction between Au­(I) and the amino group. As shown in Table S4, substituting NAC with Cys does not alter the relative order of Δ*G* for the studied reactions. Additionally, Δ*G* values were calculated using only the side chain of Cys, ethanethiol (EtSH), to further simplify the system. While EtSH ideally reduced computational complexity without significantly affecting the thermodynamic order,^22^ challenges arose in subsequent stages, particularly in locating TS. In contrast, TS were successfully identified without significant issues when Cys was used, making it the preferred model for these calculations.

Table 1 provides insights into the thermodynamic favorability of ligand exchange between the gold complexes and Cys. The Cys thiol p*K*_b_ is estimated to be 5.7 in water^42^ and significantly lower in DMSO. In fact, experimental measurements in 90% DMSO/water report values around 0.6,^43^ which is close to the estimated value of 0.9 here for DMSO. Besides the different solvents, this difference could be attributed to the choice of amides when calculating *G̅*(H^+^_(DMSO)_), leading to errors when calculating the p*K*_b_ of a thiol, such as Cys.

The extremes of Δ*G* closely correlate with the basicity of the leaving ligands. For instance, Cl^–^, a weak base with a calculated p*K*_b_ of 18.1, leads to the most endergonic reaction for Au­(IMes)­Cl, with a Δ*G* of 10 kcal/mol. In contrast, Au­(IMes)­Stzn, having Stzn as the strongest base with a calculated p*K*_b_ of 0.8, exhibits the most favorable exergonic Δ*G* of −6.0 kcal/mol. The acid–base chemistry of the ligand has a significant impact on the reaction thermodynamics, explaining the stability of Au­(IMes)­Cl.

While the basicity of the three thiolate ligands—HSpym, HSbtz, and 2tu—is relatively similar, with p*K*_b_ values of 5.8, 6.3, and 5.6, respectively, a clear correlation emerges between reactivity and hardness, in line with Pearson’s HSAB theory. The reactivity trend follows the HOMO–LUMO gap,^44^ with Stzn being the hardest ligand (6.2 eV), consistent with its higher affinity for the hard H^+^ ion, followed by 2tu (5.2 eV), and Sbtz and Spym, with very similar values (4.9 and 4.8 eV, respectively). In addition to proton affinity, the maximal interaction between the soft Au­(I)–NHC fragment and the soft Cys nucleophile further enhances ligand exchange, favoring the substitution of a hard ligand by Cys. This explains why Au­(IMes)­2tu and Au­(IMes)­Stzn undergo more extensive exchange compared to complexes with softer ligands such as HSpym. Furthermore, Au­(IMes)­(2tu) exhibits the weakest Au–S bond among the thiolates, contributing to its lower stability and increased reactivity toward Cys.

In summary, while ligand basicity emerges as the primary determinant of reaction favorability, particularly when comparing ligands with different donor atoms such as sulfur or chloride, the hardness of the ligand also plays an important role in modulating reactivity. Together, these factors highlight how both acid–base properties and the hard/soft character of the ligands govern the thermodynamics and kinetics of Au­(I)–NHC ligand exchange reactions.

### Kinetics

To evaluate the reaction kinetics, we considered three mechanisms. The NAC ^1^H NMR assay was conducted in non-dried DMSO-*d*_6_.^10^ A significant amount of water was present in the ligand exchange studies, and the H_2_O-PT (water-assisted proton transfer) mechanism was considered. Consequently, we investigated the H_2_O-PT mechanism by assuming that the water concentration in the reaction medium was sufficient to catalyze the reaction.

In the H_2_O-PT, Cys is initially deprotonated by water, forming a TS where Cys^–^ and the gold complex approach each other (TS1^H2O‑PT^) (Figure 2a). In this step, the energy of the deprotonated product was estimated combining the optimized energy of Cys^–^, the neutral water molecule, and the previously estimated solvation energy of a proton. Subsequently, Cys^–^ attacks the metal center, forming a tricoordinated transition state (TS2^H2O‑PT^). The leaving ligand dissociates in its anionic form and receives a proton from hydronium (TS3^H2O‑PT^), resulting in the formation of the thiolate product that further isomerizes to the thione form.

In the other two mechanisms, Cys remains protonated when it attacks the metal center, forming a tricoordinate transition state (TS1^SPT/NPT^). Here, the leaving ligand dissociates as an anion, which removes the proton from Cys. Depending on the specific mechanism, the proton can be abstracted by the nitrogen atom (TS2^NPT^), directly yielding the thione isomer, or by the sulfur atom (TS2^SPT^), initially producing the thioiminol isomer. For the latter, an additional isomerization step is required to achieve the most stable thione isomer. Figure 2 shows the energy variations for the reactions of all complexes with Cys reported in kcal/mol. Table 2 reports the ΔG of activation values (energies are referenced to the reactant energies set to zero) and correlates them with the LUMO energies of the complexes, the HOMO energies of the ligands (in eV), and the molecular volumes of the complexes (Å^3^).

**Table 2:** Calculated ΔG of Activation Values for TS1 and TS2 (NPT and SPT), Au­(IMes)­X LUMO Energies (eV), Molecular Volumes (Å^3^) and X HOMO Energies (in eV).

		P1 →TS2 (kcal/mol)				
complex	**R →** TS1^SPT/NPT^ (kcal/mol)	SPT	NPT	complex LUMO (eV)	X HOMO (eV)	complex volume (Å^3^)
**Au(IMes)Cl**	31.0	1.1		–0.61	–6.73	480.1
**Au(IMes)Spym**	29.5	2.7	3.8	–1.14	–5.54	571.8
**Au(IMes)2tu**	30.1	6.2	3.8	–1.15	–5.77	581.6
**Au(IMes)Sbtz**	31.5	2.8	4.6	–1.01	–5.43	625.2
**Au(IMes)Stzn**	33.1	6.1	2.8	–0.88	–5.64	576.0

These proposed mechanisms are supported by orbital calculations, which revealed significant HOMO contributions to the sulfur and nitrogen atoms of the deprotonated ligands (Figure S2). For thiopyrimidine analogs, Spym shows symmetrical HOMO coefficients on both nitrogens, while 2tu exhibits a much larger coefficient on the deprotonated nitrogen, as expected. For thiazoline derivatives, Stzn shows no HOMO electron density on the second sulfur atom, whereas Sbtz shows minimal contributions, likely due to electronic delocalization of the aromatic ring. Based on these observations, two Cys deprotonation pathways involving the leaving ligand were proposed: one mediated by nitrogen and the other by sulfur.

For Au­(IMes)­Spym and Au­(IMes)­Stzn, we were able to locate concerted TS in which Cys deprotonation and nucleophilic attack occur simultaneously. However, ΔG of these concerted TS are very close to those of the previously identified multistep TS (28.5 vs 29.5 kcal/mol for HSpym, 31.2 vs 33.1 kcal/mol for HStzn). Therefore, we have retained the original discussion focused on the multistep mechanisms while noting that these concerted TS exist but probably do not substantialy affect the energy landscape.

For the SPT pathway, a tautomerism step was necessary to convert the thioiminol intermediate to the thione product (Figure 2b—TS3^SPT^). Three tautomerization mechanisms were proposed: intramolecular proton transfer (sulfur to nitrogen), water-assisted transfer, and an intermolecular proton transfer (bimolecular). TS for these pathways were optimized using the same theoretical methodology employed in this study (Figure S3 and Table S5).

The bimolecular mechanism presented the lowest energy barrier for all complexes, followed by the water-assisted pathway. The intramolecular proton transfer mechanism has significantly higher energy barriers, likely due to the molecule adopting an unfavorable conformation for hydrogen migration. Although the water-assisted mechanism exhibits slightly lower barrier energies, its TS form six-membered rings involving sulfur, carbon, nitrogen, and water hydrogen atoms. By contrast, the bimolecular arrangement is easier for the proton transfer. As a result, the bimolecular route was incorporated into the SPT mechanism.

In the water-catalyzed mechanism, the TS2^H2OPT^ represents the Cys^–^ attack on the metal center, represented in Figure 3a for Au­(IMes)­2tu (and Figure S4 for the remaining complexes). These TS consistently display a trigonal geometry around Au­(I), in agreement with a prior study.^17^ The S–Au–S angles in TS2 are 93.5, 106.6, 82.3, 81.8, and 94.0° for Au­(IMes)­2tu, Au­(IMes)­Spym, Au­(IMes)­Sbtz, Au­(IMes)­Stzn, and Au­(IMes)­Cl, respectively.

**Figure 3:**
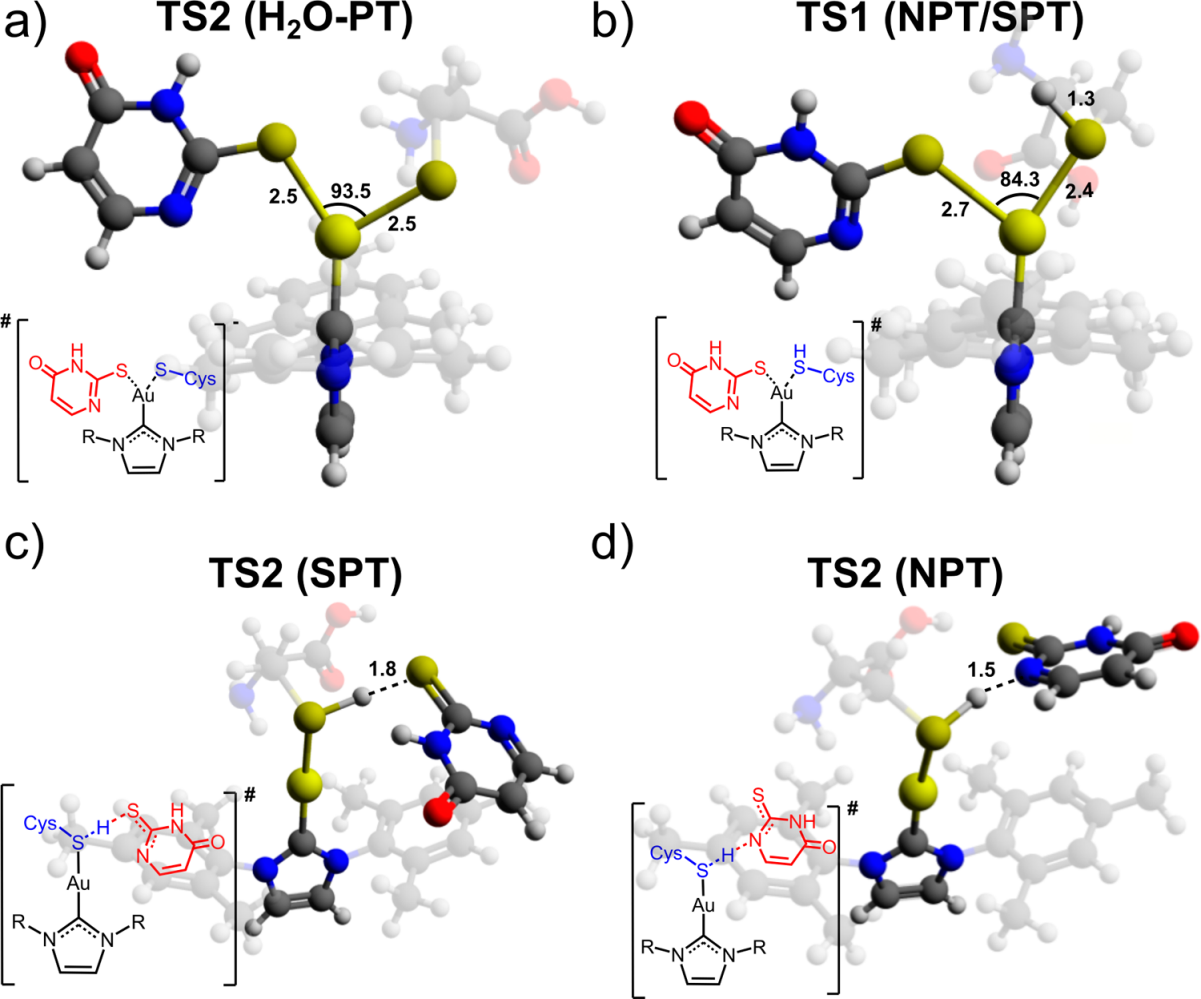
TS structures for (a) TS2^H2O‑PT^, (b) TS1^SPT/NPT^, (c) TS2^SPT^, and (d) TS2^NPT^, using Au­(IMes)­2tu as an example. Carbon, hydrogen, nitrogen, sulfur, oxygen, and gold are depicted by black, gray, blue, dark yellow, red, and yellow spheres, respectively.

The TS2^H2O-PT^ structures exhibit elongated Au–S­(Cl) bond lengths compared with the original 2.3 Å in the initial complexes. Au–Cl in Au­(IMes)Cl elongates less, from initial 2.3 to 2.4 Å. The Au–S bond lengths with Cys sulfur atoms in TS2 are 2.5 Å for Au­(IMes)­2tu, Au­(IMes)­Spym, Au­(IMes)­Sbtz, and Au­(IMes)­Stzn. The Au–S­(Cys^–^) bond lengths are 2.8 Å for Au­(IMes)­Cl, 2.6 Å for Au­(IMes)­Spym and Au­(IMes)­Sbtz, and 2.5 Å for Au­(IMes)­2tu and Au­(IMes)­Stzn.

To further investigate the SPT and NPT pathways, we analyzed the TS1^SPT/NPT^ (Figure 3b and Figure S5). Compared to the TS2^H2OPT^ structures, the spatial orientations of the Cys chain in the SPT and NPT differ significantly due to the presence of the proton. Specifically, in TS2^H2OPT^ of Au­(IMes)­Cl, Au­(IMes)­2tu, and Au­(IMes)­Spym, the S–C bond of Cys^–^ adopts an axial orientation, pointing upward. This results in substantially larger S–Au–S­(Cl) angles than those in their protonated TS1^SPT/NPT^ counterparts. For instance, in TS2^H2OPT^, the S–Au–S­(Cl) angles are 93.5, 94.0, and 106.6° for Au­(IMes)­2tu, Au­(IMes)­Cl, and Au­(IMes)­Spym, respectively, while in the corresponding protonated TS1^SPT/NPT^ structures in the NPT and SPT pathways, these angles are notably smaller: 84.0, 86.3, and 87.1°, respectively. In contrast, for Au­(IMes)­Sbtz and Au­(IMes)­Stzn, the S–C bond orientation in TS2^H2OPT^ is similar to that in protonated TS1^SPT/NPT^. Consequently, their S–Au–S angles are smaller in TS2^H2OPT^, with values of 82.3 and 81.8°, compared to the larger angles observed in their respective NPT and SPT TS1^SPT/NPT^ structures (87.1 and 87.6°).

Additionally, the Au–S­(Cys) bond lengths in TS1^SPT/NPT^ are consistently smaller than those observed in TS2^H2OPT^. The Au–S bond lengths are 2.8 Å for Au­(IMes)­Cl, 2.6 Å for Au­(IMes)­Spym, and Au­(IMes)­Sbtz and 2.5 Å for Au­(IMes)­2tu and Au­(IMes)­Stzn. The elongation in TS2^H2OPT^ is likely attributable to the larger atomic radius of sulfur in Cys^−^^−^ compared to its protonated form.

Figure 3 also highlights the TS2^SPT^ and TS2^NPT^ for hydrogen abstraction from Au­(IMes)­(CysH)^+^ by the leaving ligand, mediated by sulfur (Figure 3c) or nitrogen (Figure 3d), respectively, using Au­(IMes)­2tu as an example. The remaining TSs for this step across other complexes are listed in Figure S6.

For the SPT and NPT mechanisms, the position of the leaving ligands in the TS2 structures has an important influence on the proton transfer step and presents significant differences between them. Generally, in TS2^SPT^, the ligand approaches with its C–S bond oriented orthogonally to the S–H bond of Au­(IMes)­(CysH)^+^. In contrast, in TS2^NPT^, the ligand is positioned laterally to Au­(IMes)­(CysH)^+^, allowing hydrogen bonding between the carboxyl hydrogen of Cys and the sulfur atom of the leaving ligand, stabilizing the TS and slightly lowering the barrier.

First, the analysis of Figure 2 reveals that, in general, all complexes exhibit the same energy profiles for the proposed mechanisms. The energy barrier of the R → TS1^SPT/NPT^ (Table 2) step correlates with the electrophilicity of the complex, as indicated by the energy of its LUMO, as well as with the steric hindrance encountered during Cys attack, reflected in the complex volume. The lowest energy barrier is observed for Au­(IMes)­Spym at 29.5 kcal/mol, attributed to its stabilized LUMO, and low steric hindrance. Au­(IMes)­2tu follows closely with a barrier of 30.1 kcal/mol, where its slightly more stabilized LUMO is offset by a larger volume. Au­(IMes)Cl ranks next, with a high-energy LUMO, but less steric hindrance, compared to the thiolate ligands. Au­(IMes)­Sbtz exhibits a higher barrier (31.5 kcal/mol) due to its bulky ligand, while Au­(IMes)­Stzn has the highest barrier (33.1 kcal/mol); despite being the least bulky, it presents the least stable LUMO.

Tolbatov and co-workers found an activation free energy barrier of 40.9 kcal/mol for the reaction of an Au­(I) bis-NHC complex with Cys.^23^ Although their calculations were performed using a different level of theory, a comparison with our results suggests that the cleavage of Au–S and Au–Cl bonds in our complexes is kinetically more favorable than the cleavage of Au–C bond in bis-NHC complexes.

For the P1 → TS2^SPT^ and TS2^NPT^ step, the energy barrier depends on the nucleophilicity and steric properties, as well as stabilizing interactions in TS2. NPT is more favorable for Au­(IMes)­2tu and Au­(IMes)­Stzn with barriers of 3.8 and 2.8 kcal/mol, respectively, compared to SPT barriers of 6.2 and 6.1 kcal/mol. This is attributed to stabilizing hydrogen bonds between the sulfur atom of the leaving ligand and the Cys carboxyl group in TS2. Conversely, for Au­(IMes)­Spym and Au­(IMes)­Sbtz, the SPT is slightly more favorable due to the ligand orientation and steric effects. In Au­(IMes)­Spym, TS2^SPT^ is stabilized by a hydrogen bond with the spectator nitrogen, while in Au­(IMes)­Sbtz, steric hindrance favors sulfur deprotonation.

Au­(IMes)Cl exhibits the lowest TS2 barrier (1.1 kcal/mol), benefiting from minimal steric hindrance despite its low-energy HOMO. Au­(IMes)­Spym (2.7 kcal/mol) and Au­(IMes)­Sbtz (2.8 kcal/mol) have comparable TS2 barriers, influenced by a balance of complex volume and HOMO energy. Au­(IMes)­Stzn and Au­(IMes)­2tu have higher barriers of 6.1 and 6.2 kcal/mol, respectively, due to their lower-energy HOMO among the thiolate ligands and their larger volume. Thus, for the SPT mechanism, the order is Spym ≈ Sbtz < Stzn <2tu, while for the NPT mechanism, the order is Stzn < 2tu < Spym < Sbtz. This demonstrates how intermolecular interactions appear to balance other factors influencing the process.

The H_2_O-PT mechanism consistently shows lower barriers (TS2^H2OPT^) for the deprotonated Cys than TS1 barriers in NPT or SPT pathways, with values of 15.9, 13.6, 15.2, 22.2, and 17.3 kcal/mol for Au­(IMes)­Cl, Au­(IMes)­2tu, Au­(IMes)­Sbtz, Au­(IMes)­Spym, and Au­(IMes)­Stzn, respectively. These lower barriers reflect the higher nucleophilicity of deprotonated Cys. The order of TS2 barriers shifts compared to the protonated Cys, highlighting the influence of steric and electronic factors: Spym > Stzn > Cl > Sbtz > 2tu.

Despite the lower TS1 barriers in the H_2_O-PT mechanism, the high endergonicity of Cys deprotonation in DMSO makes this pathway unfavorable. For Au­(IMes)­Cl, the preferred route remains the deprotonation of Au­(IMes)­(CysH)^+^ over the H_2_O-PT pathway. Additionally, the NPT mechanism is generally favored for thiolate complexes due to its simpler reaction profile, avoiding the tautomerization step required in SPT, whose barriers are comparable to those of TS2.

## Conclusions

The simplified theoretical model used in this work allowed calculation of the Gibbs free energy variations for reactions of Au­(I)­NHC complexes with thiolate ligands and Cys molecules, accurately predicting the reaction extent order of Au­(I)­(IMes)­(SR) complexes and NAC, as determined experimentally using ^1^H NMR. Furthermore, the model provided additional insights into the factors governing reactivity. In addition to ligand basicity, which emerged as the dominant factor influencing reaction thermodynamics, other properties such as Au–S bond strength also played a role. The mechanism proposed starts with Cys attacking the metal center, forming a tricoordinate complex in an associative mechanism, followed by Cys deprotonation by the ligand (SR) forming the most stable tautomer, without a tautomerization step. The barriers could be rationalized based on the energy of the complexes’ orbitals and the ligands. As they are quite close, the experimental results are determined by thermodynamics. Altogether, this study highlights that subtle electronic and structural features of leaving ligands can substantially influence both the thermodynamics and kinetics of Au­(I)–Cys interactions. From a drug design perspective, this emphasizes the need to balance ligand stability with controlled reactivity.

## Supplementary Material



## Notes

Experimental p*K*_a_ of amides; geometric parameters for all complexes optimized using different functionals; optimized structures; Δ*G* for the reaction between complexes and Cys, NAC, and EtSH; molecular orbitals; tautomerism transition state structures and kinetics for the ligands (2tu, Spym, Stzn, and Sbtz); transition state structures and imaginary frequencies for [Au­(IMes)­X] (X = Spym, Sbtz, Stzn, and Cl) for TS2 (TS2^H2O‑PT^); TS1 (TS1^SPT/NPT^) and TS2 (TS2^SPT/NPT^); and xyz coordinates for all optimized structures (PDF)

## Abreviations

NHC: *N*-heterocyclic carbene
HSpym: pyrimidine-2-thione
2tuH: 2-thiouracil
HStzn: 1,3-thiazolidine-2-thione
HSbtz: 1,3-benzothiazole-2-thione
IMes: 1,3-bis­(2,4,6-trimethylphenyl)­imidazolinium
FDA: Food and Drug Administration
NAC: *N*-acetylcysteine
DFT: density functional theory
Cys: cysteine
Cys-: deprotonated cysteine
IRC: intrinsic reaction coordinate
DMSO: dimethyl sulfoxide
NMR: nuclear magnetic resonance
EtSH: ethanethiol
H2O-PT: water-assisted proton transfer
SPT: sulfur proton transfer
NPT: nitrogen proton transfer
HOMO: highest occupied molecular orbital
LUMO: lowest unoccupied molecular orbital
TS: transition state
