# Supplementary material for: Ligand Basicity Governs Cysteine Reactivity in Au(I)–NHC Thiolate Complexes: A Computational Study
Source: Inorg Chem. 2025 Sep 26;64(40):20094–102. doi: 10.1021/acs.inorgchem.5c02777 (PMC12522146; doi:10.1021/acs.inorgchem.5c02777)
Supplement: Supplementary file 1 [file ic5c02777_si_001.pdf]

SUPPORTING INFORMATION

Ligand Basicity Governs Cysteine Reactivity in Au(I)-NHC Thiolate Complexes: A Computational Study

Gustavo Clauss [a], *Igor Santos Oliveira* [a], *Camilla Abbehausen* [a]*

[a] Institute of Chemistry, State University of Campinas, Campinas, São Paulo, 13083-632, Brazil.

* Email: camilla@unicamp.br

KEYWORDS

Gold(I) complexes, Thione Ligands, Metallodrugs, Antileishmanial, Density Functional Theory (DFT)

Table S1. Calculated Gibbs Free Energy for HX and X^-, the experimental pK_A and $G(H^+_{(DMSO)})$ for 40 amides.

Molecule	Experimental pK_A DMSO[1]	$G(X^-)$ (Eh)	G(HX) (Eh)	$G(H^+_{(DMSO)})$ (kcal/mol)
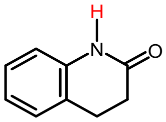	20.7	-477.776	-478.259	-274.73
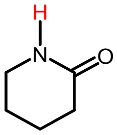	26.6	-325.367	-325.865	-276.04
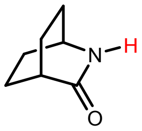	25.4	-402.743	-403.237	-275.49
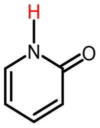	17	-323.016	-323.491	-274.54
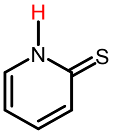	13.3	-647.560	-648.024	-273.27
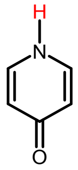	14.8	-323.013	-323.482	-274.24
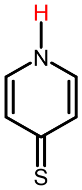	11.9	-647.562	-648.021	-271.77
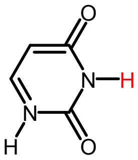	14.1	-414.403	-414.873	-275.83
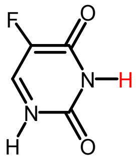	12.7	-513.743	-514.207	-273.99
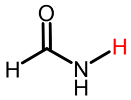	23.5	-169.444	-169.933	-275.25
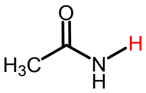	25.5	-208.727	-209.222	-275.82
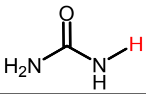	26.9	-224.816	-225.313	-275.34

Table S1. (cont.) Calculated Gibbs Free Energy for HX and X^-, the experimental pK_A and $G(H^+_{(DMSO)})$ for 40 amides.

Molecule	Experimental pK_A DMSO[1]	$G(X^-)$ (Eh)	G(HX) (Eh)	$G(H^+_{(DMSO)})$ (kcal/mol)
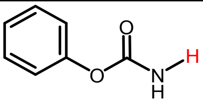	23	-514.916	-515.404	-275.10
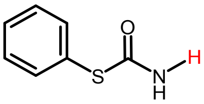	23	-839.473	-839.961	-275.08
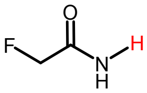	22.3	-308.070	-308.558	-275.57
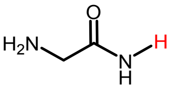	24.7	-264.074	-264.567	-275.36
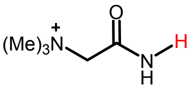	15.3	-382.331	-382.811	-280.37
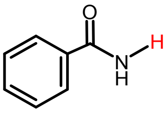	23.3	-400.388	-400.879	-275.92
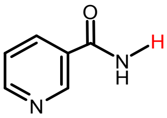	22	-416.458	-416.945	-275.58
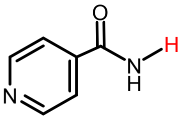	21.6	-416.459	-416.945	-275.66
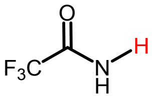	17.2	-506.791	-507.266	-274.69
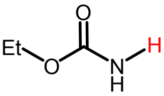	24.2	-323.278	-323.769	-275.13
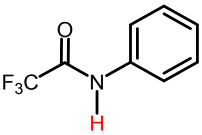	12.6	-737.729	-738.195	-274.99
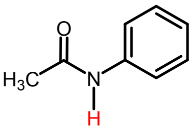	21.5	-439.668	-440.153	-274.62
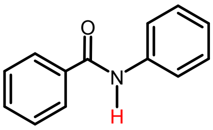	18.8	-631.330	-631.808	-274.46
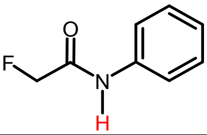	18.2	-539.009	-539.490	-276.52

Table S1. (cont.) Calculated Gibbs Free Energy for HX and X^-, the experimental pK_A and $G(H^+_{(DMSO)})$ for 40 amides.

Molecule	Experimental pK_A DMSO[1]	$G(X^-)$ (Eh)	G(HX) (Eh)	$G(H^+_{(DMSO)})$ (kcal/mol)
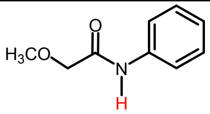	19.4	-554.187	-554.667	-274.89
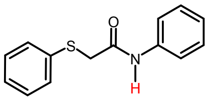	19	-1070.411	-1070.890	-274.89
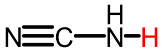	17	-148.337	-148.804	-269.90
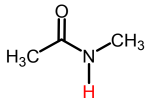	25.9	-247.996	-248.491	-275.71
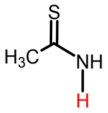	18.5	-533.269	-533.748	-274.78
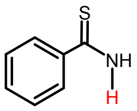	16.9	-724.929	-725.405	-275.89
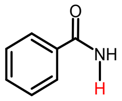	23.3	-400.388	-400.879	-275.84
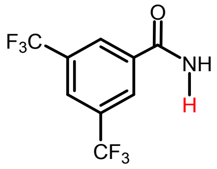	16.9	-1075.065	-1075.551	-282.40
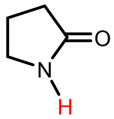	24.2	-286.095	-286.587	-276.04
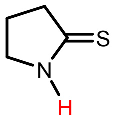	13.3	-610.640	-611.117	-281.18
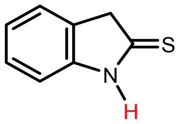	10	-763.040	-763.506	-279.00
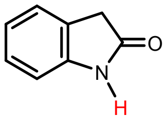	18.5	-438.501	-438.978	-274.13
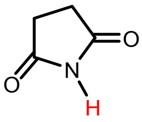	14.7	-360.230	-360.698	-273.59
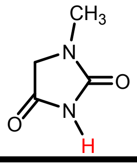	15	-415.582	-416.050	-273.35

Table S2. Comparison of crystal and calculated structures of [Au(IMes)X], X = 2tu, Spym, Sbtz, Stzn and Cl.

Parameter	[Au(IMes)(2tu)]				
	**Exp.[2]**	**PBE0**	**ωB97X**	**B3LYP**	**M06-2X**
Au-C (Å)	1.989(3)	2.004	2.004	2.028	2.012
Au-S (Å)	2.2915(7)	2.3065	2.3077	2.3347	2.3525
S-C (Å)	1.745(3)	1.736	1.739	1.747	1.740
N-C (Å)	1.354(4)	1.348	1.342	1.355	1.345
C-Au-S (°)	179.98(8)	177.92	179.36	175.31	179.04
RMSD (Å)	N/A	0.7818	0.6311	0.8997	0.6060
**Parameter**	**[Au(IMes)(Spym)]**				
	**Exp.[2]**	**PBE0**	**ωB97X**	**B3LYP**	**M06-2X**
Au-C (Å)	1.992(3)	2.007	2.006	2.031	2.016
Au-S (Å)	2.2849(8)	2.3013	2.3036	2.3301	2.3411
S-C (Å)	1.755(3)	1.750	1.753	1.762	1.755
N-C (Å)	1.352(4)	1.348	1.344	1.355	1.345
C-Au-S (°)	170.99(8)	177.39	179.15	176.09	179.22
RMSD (Å)	N/A	0.7223	0.7911	0.6915	0.8190
**Parameter**	**[Au(IMes)(Sbtz)]**				
	**Exp.[2]**	**PBE0**	**ωB97X**	**B3LYP**	**M06-2X**
Au-C (Å)	1.999(3)	2.008	2.007	2.030	2.016
Au-S (Å)	2.2935(8)	2.3040	2.307	2.335	2.352
S-C (Å)	1.744(3)	1.732	1.739	1.744	1.741
N-C (Å)	1.349(3)	1.348	1.343	1.354	1.345
C-Au-S (°)	176.59(7)	178.61	178.71	178.04	177.12
RMSD (Å)	N/A	1.0030	0.2435	1.0397	0.2242
**Parameter**	**[Au(IMes)(Stzn)]**				
	**Exp.[2]**	**PBE0**	**ωB97X**	**B3LYP**	**M06-2X**
Au-C (Å)	2.001(5)	2.010	2.007	2.032	2.016
Au-S (Å)	2.2964(10)	2.3042	2.3068	2.3347	2.3466
S-C (Å)	1.736(11)	1.742	1.746	1.755	1.751
N-C (Å)	1.348(7)	1.348	1.343	1.355	1.345
C-Au-S (°)	179.32(18)	177.37	179.65	176.95	179.34
RMSD (Å)	N/A	0.7152	0.4839	0.7735	0.5237
**Parameter**	**[Au(IMes)Cl]**				
	**Exp.[3]**	**PBE0**	**ωB97X**	**B3LYP**	**M06-2X**
Au-C (Å)	1.999(5)	2.018	1.983	2.001	1.985
Au-Cl (Å)	2.2758(12)	2.3212	2.3025	2.3305	2.3538
N-C (Å)	1.337(4)	1.345	1.343	1.355	1.346
C-Au-Cl (°)	180.0	178.7	179.6	179.7	179.3
RMSD (Å)	N/A	0.2062	0.1328	0.2269	0.1934

Table S3. ΔG (kcal/mol) values for the reaction of [Au(IMes)X], X = 2tu, Spym, Sbtz, Stzn and Cl, and Cys, calculated using different functionals.

**Complex**	**ΔG (kcal/mol)**				
	**Exp.**	**PBE0**	**ωB97X**	**B3LYP**	**M06-2X**
Au(IMes)2tu	-3.8	-3.9	-3.7	-2.4	-7.6
Au(IMes)Spym	-0.6	2.8	2.2	0.8	-0.5
Au(IMes)Stzn	-1.5	-2.4	-0.3	1.5	-3.4
Au(IMes)Sbtz	-5.4	-6.0	-4.2	-5.8	-7.9
Au(IMes)Cl	2.5	10.0	6.2	7.3	3.7
RMSE (kcal/mol)	N/A	3.7	3.6	4.3	2.3

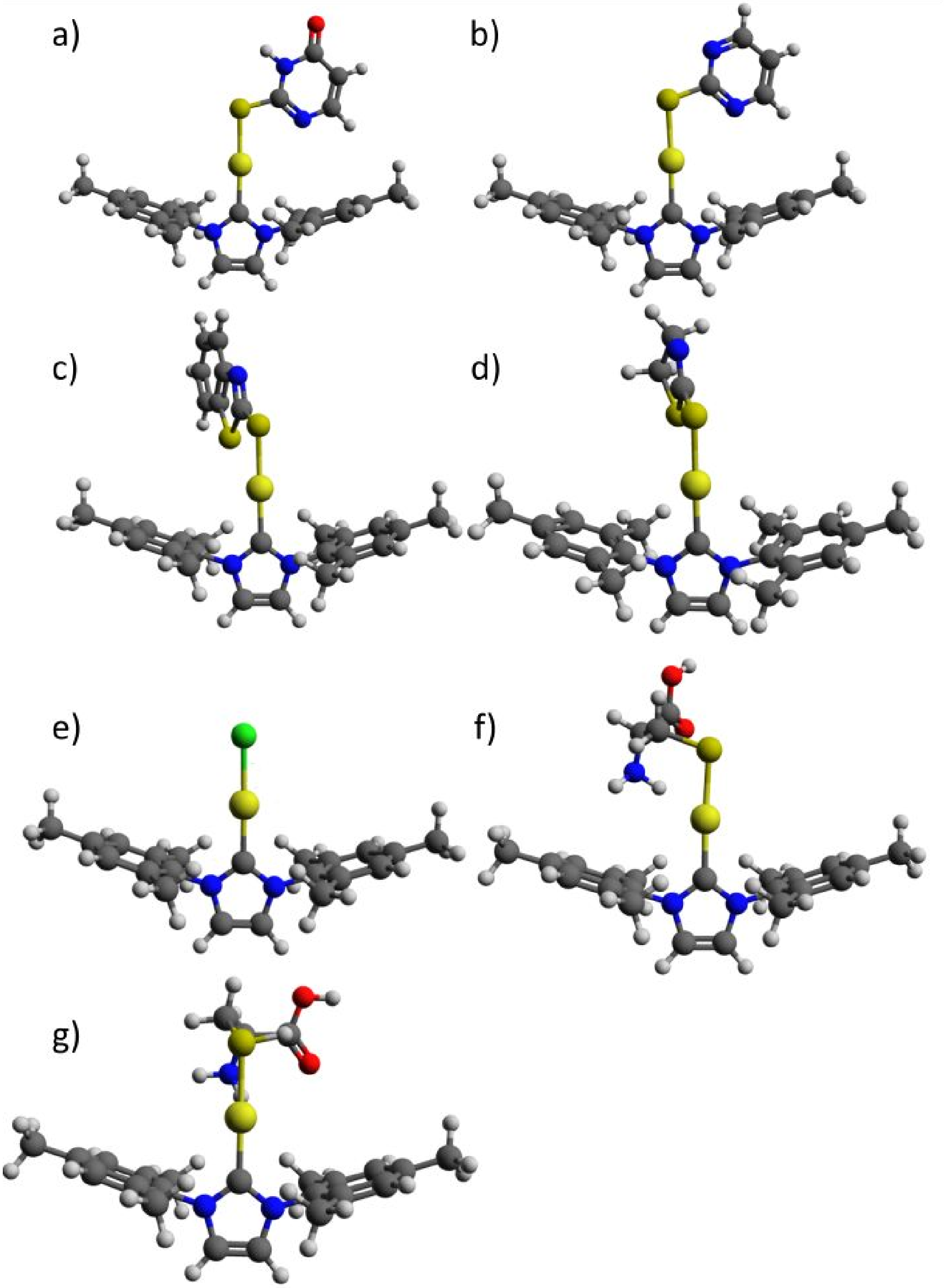

Figure S1. [Au(IMes)X] optimized structures, X = **a)** 2tu, **b)** Spym, **c)** Sbtz, **d)** Stzn, **e)** Cl, **f)** Cys^{-} and **g)** Cys. The structures were optimized using PBE0, SARC-def2-TZVP for Au(I), ZORA-def2-TZVP for other atoms, SARC/J and CPCM for DMSO implicit solvation.

Table S4. Gibbs Free Energy variation for the reaction of [Au(IMes)X] with Cys, NAC and EtSH.

**Complex**	**% Bound NAC experimental**	**ΔG (kJ/mol)**		
		**NAC**	**CysH**	**EtSH**
**[Au(IMes)Cl]**	11	8.6	10.0	8.9
**[Au(IMes)(2tu)]**	96	-5.2	-3.8	-5.0
**[Au(IMes)(Spym)]**	62	1.4	2.8	1.7
**[Au(IMes)(Sbtz)]**	78	-3.7	-2.3	-3.5
**[Au(IMes)(Stzn)]**	100	-7.3	-6.0	-7.1

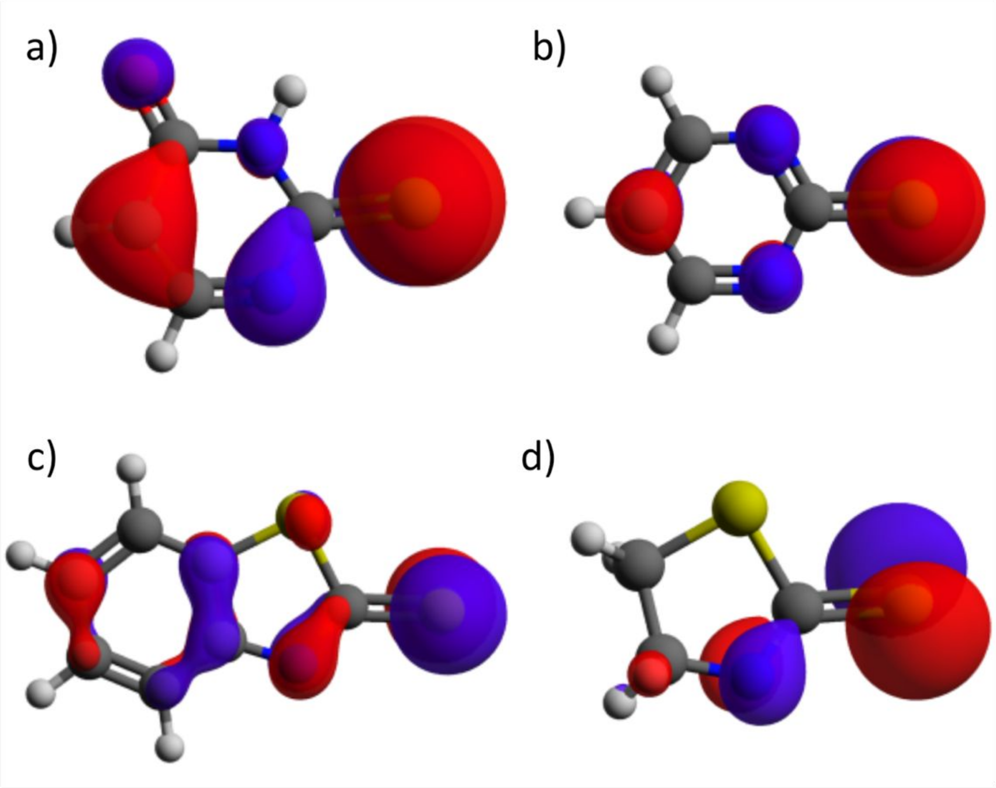

Figure S2. HOMO orbitals for **a)** 2tu, **b)** Spym, **c)** Sbtz and **d)** Stzn. Calculated using PBE0, ZORA-def2-TZVP, SARC/J and CPCM for DMSO implicit solvation.

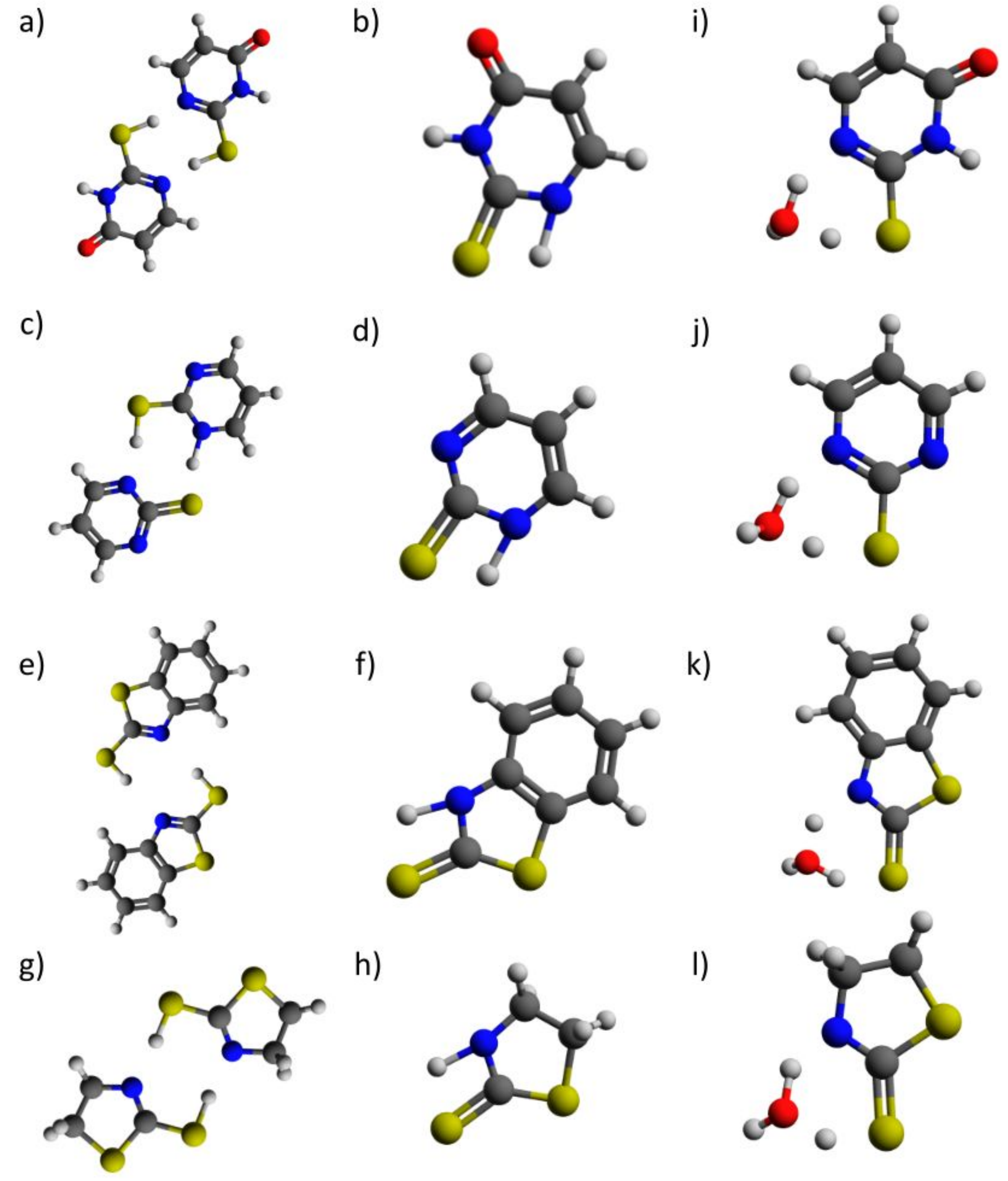

Figure S3. Tautomerism transition state structures considering bimolecular (a, c, e and g), direct proton transfer (b, d, f and h) and H_2O assisted mechanisms (i, j, k and l) for 2tuH, SpymH, SbtzH and StznH, respectively.

Table S5. Tautomerism mechanisms (Thioiminol → Thione) Gibbs Free Energy of activation (ΔG‡) for all molecules.

Molecule	ΔG‡ (kJ/mol)		
	**Direct proton transfer**	**Bimolecular**	**H_2O Assisted**
**2tu**	96.8	37.4	42.0
**Spym**	108.3	53.5	60.7
**Sbtz**	120.5	55.7	41.2
**Stzn**	112.2	52.5	57.3

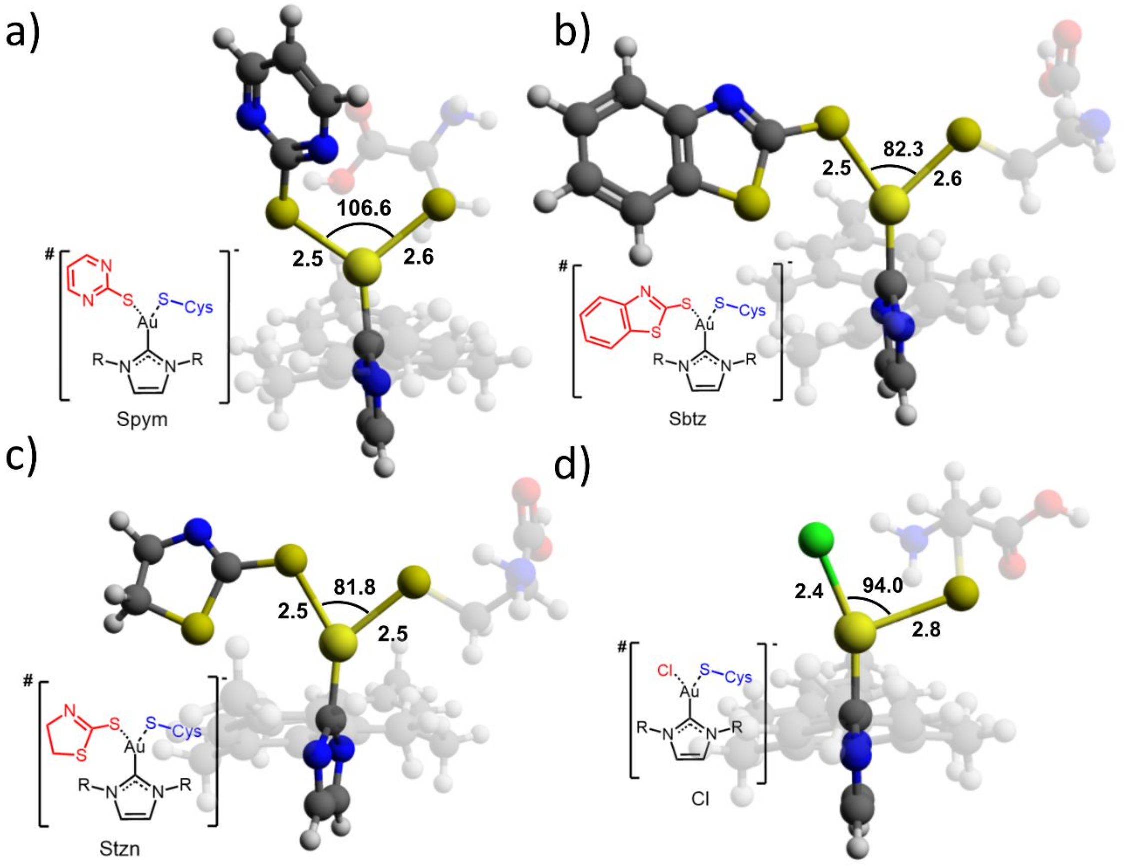

Figure S4. TS2$^{H2O\text{-}PT}$ structures for [Au(IMes)X], X = **a)** Spym, **b)** Sbtz, **c)** Stzn and **d)** Cl.

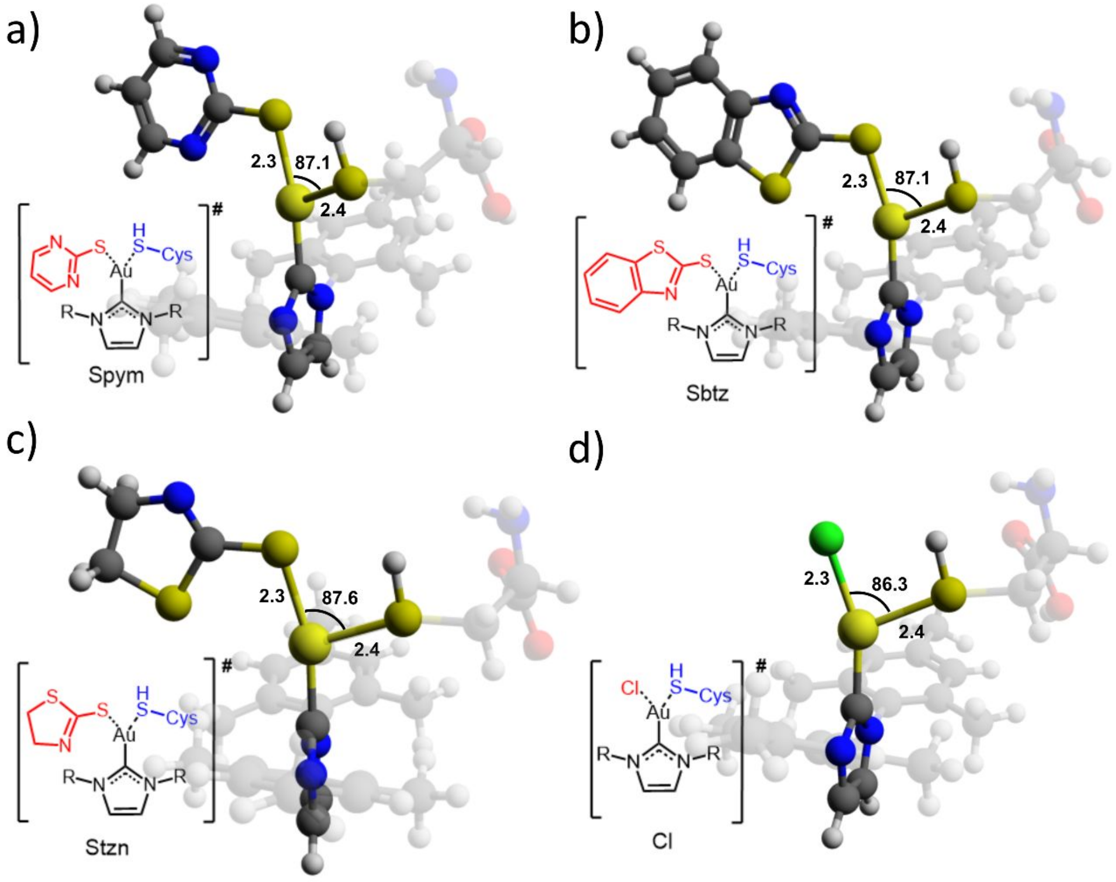

Figure S5. TS1$^{SPT/NPT}$ structures for [Au(IMes)X], X = **a)** Spym, **b)** Sbtz, **c)** Stzn and **d)** Cl.

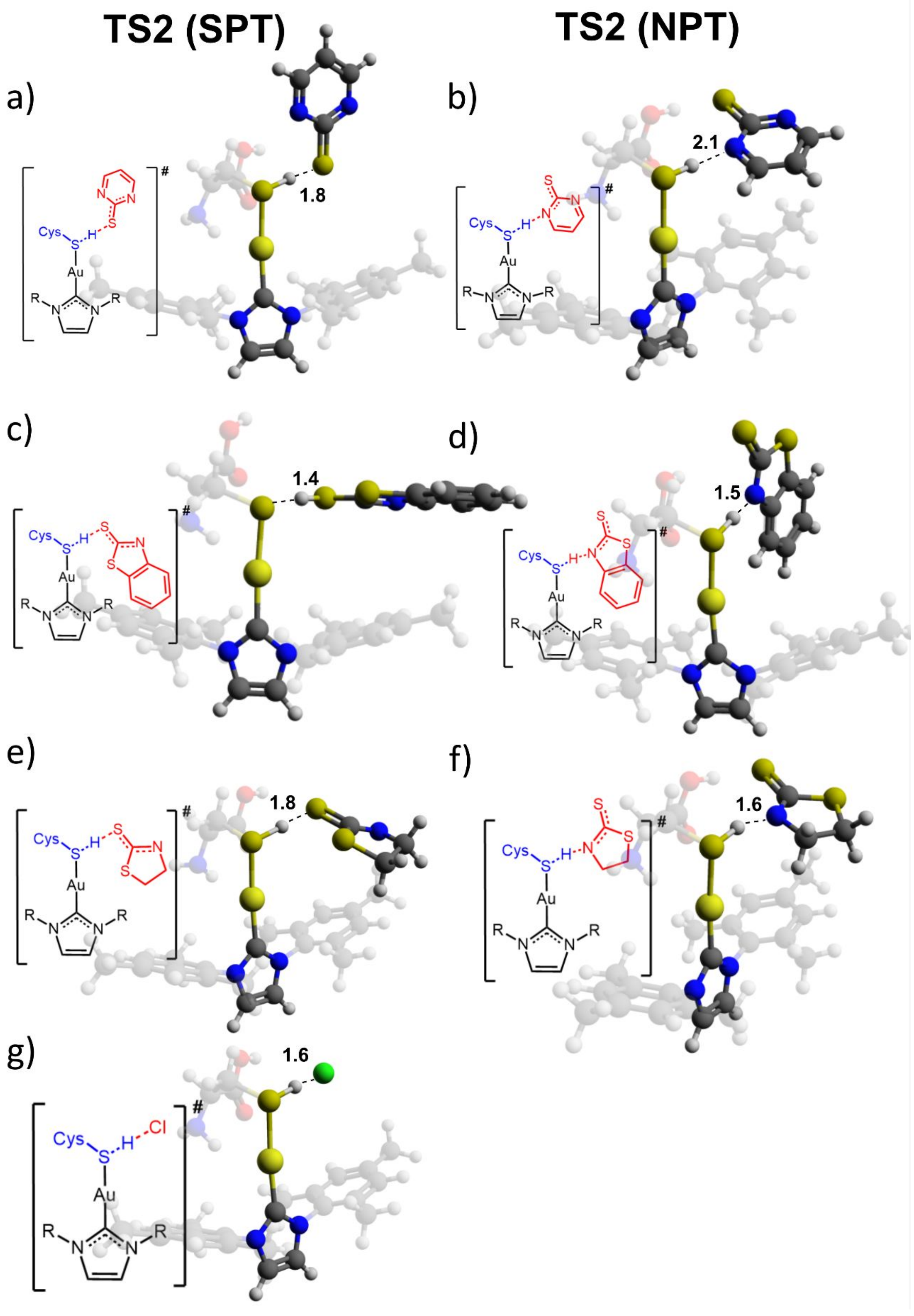

Figure S7. TS2$^{SPT/NPT}$ structures for [Au(IMes)X], **a,b)** Spym, **c,d**) Sbtz, **e,f**) Stzn and **g)** Cl.

Table S6. Imaginary frequencies (cm^{-1}) for the obtained transition state (TS) structures.

TS	Au(IMes)X				
	**2tu**	**Spym**	**Sbtz**	**Stzn**	**Cl**
$TS1^{H2O-PT}$	-245.16	-245.16	-245.16	-245.16	-245.16
$TS2^{H2O-PT}$	-18.24	-34.68	-29.00	-31.59	-27.21
$TS3^{H2O-PT}$	-1037.75	-134.58	-887.70	-160.02	-462.67
$TS1^{SPT/NPT}$	-37.96	-41.42	-40.09	-62.90	-55.55
$TS2^{SPT}$	-416.95	-563.58	-347.17	-375.09	-496.44
$TS3^{SPT}$	-147.58	-1006.65	-465.92	-571.70	N/A
$TS2^{NPT}$	-610.78	-70.03	-609.09	-134.64	-496.44

Table S7. Coordinates (x,y,z) of optimized structures of reactants, products and transitions states.

2tuH

Coordinates from ORCA-job

N	6.02530638634962	-1.42846948963182	4.22180862423943
C	7.17807262920815	-0.94054248199875	4.75609671491221
C	5.68020580056187	-2.73689083716661	4.26063941928559
N	6.58561848235863	-3.53568975785689	4.87249523558249
C	7.79878967934575	-3.15512544083528	5.45060054443771
C	8.07012565510867	-1.74384470157867	5.36341590334999
O	8.50976544154362	-3.99994115461374	5.96683766641437
S	4.25892907584292	-3.30843280430250	3.60794602315134
H	7.31638169090938	0.12817745347166	4.65441650711328
H	8.98411264287415	-1.35599153093180	5.78762009992086
H	6.35897765315076	-4.52105521108565	4.91826347354225
H	5.37385486274644	-0.79909404346993	3.77578978805044

SpymH

Coordinates from ORCA-job

N	5.98867085522480	-1.48841533235583	4.34407023395595
C	7.04405864457265	-0.95568921223188	4.97250813293594
C	5.67068996600118	-2.82305444699221	4.37947875864603
N	6.47796314081406	-3.63826486147583	5.09350231739900
C	7.51825883894159	-3.12632397265713	5.71552745706953
C	7.86454834209092	-1.76986475346385	5.69600142788271
H	8.13134552367676	-3.82897292692222	6.27425062037777
S	4.31839364934150	-3.36884474903526	3.54982963248284
H	7.18128593680695	0.11260446140888	4.86421273477344
H	8.72726540340930	-1.38618305829253	6.21965555040777
H	5.37476969912026	-0.88800114798213	3.80754313406901

SbtzH

Coordinates from ORCA-job

C	5.04701467383541	-5.30667306798380	5.73825002106311
C	4.09471694763452	-4.36649837074587	6.13827447566564
N	5.41558715568799	-5.13456918360102	4.41996163549642
S	3.72008677723159	-3.30896804065253	4.80947211790392
C	4.82833752774348	-4.12987520371755	3.74820546778828
S	5.08902457237989	-3.70871209335501	2.16421380850062
C	5.51858265497212	-6.26757583078739	6.62157108913686
C	5.01430322469364	-6.26526372772408	7.91135040811251
C	4.06229095072492	-5.32851522773442	8.31301276740297
C	3.59153570169837	-4.36874952838752	7.43028012082519
H	3.68334528826252	-5.34893439943982	9.32795261815236
H	2.85204156486496	-3.63987363746684	7.73915843007677
H	6.25808353045689	-6.99339439039765	6.30434117059075
H	5.36764894026079	-7.00577015743674	8.61927813987074
H	6.09481048955291	-5.72685714056973	3.96066772941383

StznH

Coordinates from ORCA-job

C	5.10705598051754	-5.25832943539573	5.90803210538420
C	3.85825610513338	-4.44605386567749	6.20841730114851
N	5.24100452726917	-5.25123674957801	4.46314839072037
H	5.01337426236057	-6.28199187510137	6.27125955244306
H	5.99322307170129	-4.80238246901143	6.36216646143671
S	3.77898396881900	-3.22287804683213	4.86826137538906
H	3.91885218291933	-3.93624022640993	7.16785526063290
H	2.95541059528216	-5.05606738245819	6.17323002946399
C	4.73886205427567	-4.21706701141089	3.80252609200830
S	4.96942088358387	-3.85611278262132	2.19489473211696
H	5.88456636813800	-5.87380015550348	3.99575869925591

2tu

Coordinates from ORCA-job

N	6.036527	-1.375849	4.221786
C	7.201197	-0.977391	4.772487
C	5.750140	-2.671316	4.285524
N	6.630482	-3.525467	4.893699
C	7.838126	-3.179422	5.478811
C	8.113337	-1.783189	5.391142
O	8.539570	-4.051026	5.998793
S	4.318494	-3.336499	3.635080
H	7.400535	0.089651	4.699668
H	9.030100	-1.394919	5.813358
H	6.379162	-4.504352	4.921413

Spym

Coordinates from ORCA-job

N	5.976072	-1.435472	4.330069
C	7.038767	-0.977925	4.975027
C	5.716844	-2.763364	4.402699
N	6.491975	-3.623984	5.104934
C	7.546165	-3.129687	5.736960
C	7.886474	-1.787490	5.712573
H	8.152005	-3.843320	6.292078
S	4.343729	-3.383191	3.562652
H	7.223418	0.091954	4.898836
H	8.751812	-1.399232	6.232532

Sbtz

Coordinates from ORCA-job

C	5.047029	-5.300236	5.732582
C	4.084107	-4.359931	6.157962
N	5.461858	-5.188822	4.427949
S	3.720211	-3.318392	4.826171
C	4.870298	-4.204359	3.801327
S	5.082543	-3.713945	2.184523
C	5.502391	-6.256737	6.642354
C	5.001403	-6.261765	7.933318
C	4.048952	-5.325433	8.338620
C	3.582057	-4.365704	7.451792
H	3.668443	-5.345897	9.353554
H	2.841842	-3.637850	7.764159
H	6.242128	-6.984592	6.327209
H	5.354638	-7.004577	8.640171

Stzn

Coordinates from ORCA-job

C	5.073572	-5.244761	5.891035
C	3.825001	-4.422336	6.225904
N	5.312236	-5.245593	4.460225
H	4.965643	-6.269095	6.258674
H	5.950574	-4.814395	6.394306
S	3.762687	-3.194268	4.905010
H	3.896605	-3.938824	7.200009
H	2.918546	-5.030232	6.195186
C	4.779270	-4.251330	3.854884
S	4.936725	-3.796586	2.208075

Au(IMes)2tu

Coordinates from ORCA-job

H	6.99415749094694	1.63023190433623	-0.03264551462690
C	6.24543430901801	1.77373174442224	0.74038053416116
C	4.60262091956808	0.62093030661636	-0.76917671945326
C	4.95489507109155	1.30918438462717	0.51339780882445
Au	3.28570266292589	-1.64566869602738	2.54753833948220
H	-0.92580399240133	-4.02764476586573	-0.47512987869563
C	-0.83255418182583	-3.35028137555402	0.36821622392218
C	8.00514136067731	2.87150353573023	2.15792258032703
C	6.59760726436774	2.41454071958118	1.92355287016299
C	-1.54230343609298	-3.60957464746706	1.53595659471410
C	-2.40210068118760	-4.83104065708926	1.65351004850033
C	-0.00087132102351	-2.24348631502718	0.24586991437183
C	0.75495935042158	-1.98978942897473	-1.02139793432790
C	-1.41496147273356	-2.72218756989023	2.59918096295843
C	0.10473360390128	-1.38609878852498	1.34094738849598
C	2.24291866153302	-0.20372763120547	1.62517876294999
C	4.00923134991093	1.50363939398502	1.52002922603294
C	-0.59718913345788	-1.59967177902087	2.52646486501277
C	5.61859372385033	2.59920341396900	2.89418784073517
H	-1.96940054564354	-2.90360963718499	3.51486985158578
N	0.95484688493766	-0.23920810487508	1.23048938161407
N	2.67402845732766	1.03263490644811	1.30596003200793
C	4.31458107593470	2.15040259298548	2.71695865734397
C	-0.47938495847548	-0.66091503641721	3.68702046734199
C	0.58173983442500	0.96397067895470	0.66983895952108
C	1.66563257915758	1.76608542989371	0.71770161671854
H	5.87386985075859	3.10633283042737	3.81968098732626
H	-0.41201229374224	1.13492205339515	0.29139860326462
H	1.81307383571851	2.78186969374199	0.39147623286313
C	3.28317968534385	2.34722779823430	3.78429377184555

H	0.54800461458321	-0.62071018383208	4.05952344630499
H	-1.12696657213007	-0.98183590630195	4.50278636385918
H	-0.75843890100502	0.35829003959859	3.40690807474635
H	-2.81872532443251	-5.11699033200544	0.68611987336792
H	-3.22298700749020	-4.67309286062667	2.35507236375541
H	-1.81562102711305	-5.67928901751141	2.02073645229382
H	0.58006479180058	-2.79419758633669	-1.73565029069000
H	1.83018562303122	-1.92138136764093	-0.83543987187538
H	0.45001455790562	-1.04885669018154	-1.48721378972938
H	2.41028326381378	2.88493044166403	3.40584658757623
H	3.70022652153754	2.91347654317524	4.61696313600312
H	2.92663780114328	1.38694216375054	4.16792461182863
H	8.50571643087339	3.11295377767168	1.21867509280676
H	8.58792973240791	2.08388017757114	2.64640412505282
H	8.03486155286784	3.74875405717363	2.80650059485431
H	5.47015873387355	0.57685946939408	-1.42737560677608
H	3.79909745308258	1.14224520379001	-1.29610954686848
H	4.25700571324006	-0.40074868076356	-0.58890442189068
N	6.07623784167457	-1.30382167364578	4.17908613385028
C	7.19671738798923	-0.84697717689997	4.79062931197980
C	5.80082376534395	-2.57626351068930	4.33291246829722
N	6.60160360551324	-3.39055819144543	5.06897067793010
C	7.76316496923812	-2.98943119587267	5.72087726739305
C	8.04499692179953	-1.59998293378829	5.54087780781461
O	8.41836548851184	-3.80582254067246	6.36276668906947
S	4.41366317345916	-3.34455690449888	3.62523956696853
H	7.39851688829542	0.21089132695568	4.64566843679345
H	8.92619359234433	-1.17919546851129	6.00532748765172
H	6.35134245260854	-4.36691793374429	5.16321448265137

Au(IMes)Spym

Coordinates from ORCA-job

H	7.09615880079029	1.53121858078969	-0.02691209450999
C	6.33102775541560	1.68436844237588	0.72797998097903
C	4.71105009412390	0.54189189583045	-0.81526630500002
C	5.04184956691437	1.23049601591006	0.47277752220035
Au	3.28317635958778	-1.69530407848457	2.51614868093421
H	-0.99178272908877	-4.10600116539617	-0.30050920294506
C	-0.88748376415592	-3.36686523379643	0.48791425238489
C	8.06599950308950	2.77468561744941	2.18254779110418
C	6.66113714665132	2.32687012102995	1.91665156054754
C	-1.62941909556278	-3.50395905092543	1.65631232002184
C	-2.54012867266408	-4.67741352743925	1.85117184165700
C	-0.00972252029644	-2.30604864539486	0.29577387085785
C	0.78393134394523	-2.18762113937892	-0.96842184459273
C	-1.48562938264206	-2.54116730784061	2.64986899797005
C	0.10841238809264	-1.36852073639624	1.32127113156936
C	2.28067760527019	-0.24080554968603	1.56243784918355
C	4.07483648682637	1.43663251840684	1.45614676545401
C	-0.62146918809427	-1.46188779329531	2.50594836122384
C	5.66151995836279	2.52325817350895	2.86364960270001
H	-2.06290741502619	-2.62833563484563	3.56524593750059
N	0.99844020894528	-0.26119949528638	1.14681236961625
N	2.74045457715351	0.97799990883246	1.21484744247200
C	4.35823499622910	2.08511771317743	2.65771924352588
C	-0.47771091550994	-0.44374535415817	3.59412579800999
C	0.65817219836071	0.93211720623705	0.54548237946382
C	1.75696573063858	1.71377011737474	0.58819007703547
H	5.89946939861577	3.03233167260515	3.79270740854522
H	-0.32627831801899	1.11169980832531	0.14716703880154
H	1.92982086394230	2.71650036892847	0.23514244831985
C	3.30412729645368	2.29577345684307	3.70015024922256

H	0.54135866300705	-0.43501929417407	3.99108693066691
H	-1.16193501832282	-0.66240796375939	4.41382740713077
H	-0.68826352631143	0.56535485296683	3.23052882881431
H	-2.93474322572597	-5.03516883604222	0.89861269851468
H	-3.37712506208594	-4.42609626404447	2.50492011241805
H	-2.00030383541987	-5.50922459409230	2.31491495446746
H	0.56461466326201	-3.02227519847782	-1.63407043522461
H	1.85783412318575	-2.18498230926000	-0.76233853630111
H	0.55816270124796	-1.25915027228366	-1.49973913351422
H	2.44145636320359	2.83252488540915	3.29751161438885
H	3.70461213214292	2.86851982405931	4.53652670280285
H	2.93580424010534	1.34042266293180	4.08491975574782
H	8.59121041486666	3.00689353131213	1.25451188029683
H	8.63083914000957	1.98618867420389	2.69024166336348
H	8.08607049802233	3.65583828949326	2.82624099517775
H	5.59760046346502	0.46562344998531	-1.44459645749737
H	3.94214353058514	1.08389749393715	-1.37226172315704
H	4.32742846949209	-0.46669020333928	-0.63848808353369
N	5.97793272818844	-1.42228979913595	4.32510697918501
C	7.04400367107624	-0.96869886364846	4.97548761632522
C	5.73491470429183	-2.73544362411739	4.40755121460622
N	6.48170842122519	-3.61321972579086	5.09080766800811
C	7.53935700456322	-3.13213535636798	5.73121528477408
C	7.87956917165003	-1.79013849747716	5.71106159065228
H	8.13848364405900	-3.85224183926306	6.28259837170122
S	4.35405936220590	-3.42850851656395	3.58629926208586
H	7.23300017595285	0.09928785735356	4.90148279172607
H	8.74422610364017	-1.40832726903639	6.23674857210153

Au(IMes)Sbtz

Coordinates from ORCA-job

H	6.70501383914286	1.83454940322457	-1.44189912711155
C	6.24615284675835	1.79922931321687	-0.45861529059892
C	4.15331247925406	0.96210954299358	-1.56719109265549
C	4.92725796383467	1.37131466475403	-0.35213873527139
Au	3.78711457935070	-1.93475465177680	1.76718867965963
H	-1.01906798664625	-4.07228950988864	0.14138085115585
C	-0.71979453282219	-3.39908824716664	0.93890675529657
C	8.41684301212673	2.60468505364391	0.51851678561144
C	6.98643427517416	2.18001459986947	0.65508111456330
C	-1.13847223565585	-3.65302173256425	2.24011905484867
C	-1.96412603899716	-4.86163324405636	2.56086073845557
C	0.07626546784090	-2.30264269900349	0.62380519332338
C	0.52055606329843	-2.05793335641755	-0.78492194777458
C	-0.75474256308547	-2.76969253847258	3.24458859704362
C	0.44734072092789	-1.45213162995994	1.66377728572519
C	2.62173048097539	-0.35033551202827	1.36069178637249
C	4.35795868407071	1.33641429419022	0.91998112963351
C	0.03886648286541	-1.65982027633280	2.98174757500247
C	6.37465891514177	2.13858225265220	1.90376382331095
H	-1.08005602640521	-2.94741003766728	4.26513505108330
N	1.27441312143079	-0.32030878164459	1.37020822076999
N	2.99870129259193	0.90575494271499	1.05170821247000
C	5.05844883404047	1.72067876642559	2.06347060764862
C	0.44338466735230	-0.72739797764639	4.08109285414036
C	0.81038180342800	0.94175508648350	1.07054880224382
C	1.89822687408511	1.71484983729153	0.86794768845977
H	6.93417296262534	2.44405911321791	2.78269093978344
H	-0.24127512194195	1.17083538681182	1.02982029361160
H	1.99047444834406	2.75737501129112	0.61363021845694
C	4.42807651518321	1.68026305906829	3.42100227970089

H	1.53147627132826	-0.67460214375731	4.17555462420252
H	0.03375287184366	-1.06304809719231	5.03362682994481
H	0.08756018770890	0.28933916516766	3.89473198951763
H	-2.46315546487334	-5.24858639832826	1.67131077566170
H	-2.71978517529083	-4.63615671140260	3.31598462734844
H	-1.33416502517032	-5.66172439627716	2.96215733662809
H	0.09115688728274	-2.80317208665965	-1.45426829116131
H	1.60987807863458	-2.10885383577332	-0.86930356140739
H	0.21817202418284	-1.06738122632146	-1.13456836391965
H	3.47072146857794	2.20680775178938	3.43696173777514
H	5.08590439726569	2.13864918338883	4.15932036257458
H	4.23286470073905	0.64982246305912	3.73272000091858
H	8.62648546917563	2.97513761565612	-0.48607862344721
H	9.08822444464636	1.76075961294574	0.70570707916808
H	8.66826868529390	3.38504815265725	1.23917027888730
H	4.78617913243909	1.00577581953215	-2.45339835670218
H	3.29224684041332	1.61543142954449	-1.73136613837665
H	3.76823760590558	-0.05643868402954	-1.46841621534695
C	5.04143448942954	-5.29646550870863	5.73375369371100
C	4.08083503169642	-4.35851612531056	6.15997849595256
N	5.44857220397225	-5.17399043873241	4.42424057783103
S	3.70848066540479	-3.30750078838813	4.83516528726809
C	4.84543290911390	-4.18940668984129	3.83346159794129
S	5.12822334745569	-3.76248367439724	2.17883903213145
C	5.50355778630093	-6.25563449946518	6.63536955803128
C	5.00478828277007	-6.26150142568818	7.92556984007215
C	4.05125559260618	-5.32577552045530	8.33394864600130
C	3.57920655209166	-4.36446396505658	7.45459290399029
H	3.67402925747167	-5.34981286468185	9.34987801227800
H	2.83942467579619	-3.63804003674923	7.77006741581062
H	6.24338183008423	-6.98064502758515	6.31523740735183
H	5.35928215143260	-7.00400118223602	8.63159309440156

Au(IMes)Stzn

Coordinates from ORCA-job

H	6.80043693879642	1.86655330590843	-1.12841381628091
C	6.26433140893058	1.80793556307690	-0.18622107378304
C	4.28387697445313	0.95330818542781	-1.47344321630275
C	4.94936395834094	1.35477807887109	-0.19328849847834
Au	3.65370876210414	-2.00177114695491	1.77342625999451
H	-1.10823477076417	-4.06000134369461	-0.24146778585380
C	-0.84104588959205	-3.41198246148823	0.58776931151728
C	8.33105338468734	2.64098318807075	0.97621869211721
C	6.90386643200793	2.18511479127493	0.98919976540614
C	-1.33685000710301	-3.68757516953907	1.85843298563251
C	-2.20339760638341	-4.88652593467754	2.09606346276364
C	-0.00959433139993	-2.32550634666505	0.34438602604522
C	0.51743953191620	-2.05539390459414	-1.03070306408024
C	-0.99298562211148	-2.83729249518139	2.90328565296190
C	0.31856443853931	-1.50615205810810	1.42473360178535
C	2.51696085783439	-0.41058933337900	1.30890267710782
C	4.27935393183751	1.28922272816632	1.02735429452518
C	-0.16559223239885	-1.73604028531541	2.71146113856143
C	6.19398572167798	2.11081544593095	2.18361015247345
H	-1.37967693441270	-3.03153242289076	3.89894637407219
N	1.17324128428230	-0.37881090403986	1.20399379752680
N	2.91737205218982	0.84823598966649	1.03978414796603
C	4.87825872336277	1.66605242485819	2.23002237048176
C	0.19273573040805	-0.83650540576580	3.85345413290235
C	0.73592811064709	0.88443559461206	0.87116801378697
C	1.83650122965493	1.65842676092917	0.76628356733757
H	6.67482648652791	2.41176560926356	3.10948999540856
H	-0.30864394796734	1.11339351725491	0.74301413964821
H	1.94996655556521	2.70238376548874	0.52704119914547
C	4.14195557629949	1.59167872533810	3.53148105467790

H	1.27173934500419	-0.83520013841325	4.03107726345986
H	-0.30311138108057	-1.16470900801507	4.76674696104602
H	-0.10111905952884	0.19767263017292	3.65630515486979
H	-2.87967352303631	-5.05909430004838	1.25654339509917
H	-2.79574256051108	-4.77293614820881	3.00515805301985
H	-1.59162620671128	-5.78699701274755	2.20977246589735
H	0.12839737128705	-2.78755933164691	-1.73806203796785
H	1.60982094145111	-2.10552337003492	-1.05109369974483
H	0.23605906507644	-1.05836950662417	-1.37958186853447
H	3.18160811452738	2.11092997609568	3.48166715155481
H	4.73461539833706	2.03927545830117	4.32919790707169
H	3.93177953278465	0.55341342558764	3.80448026535712
H	8.62627827544584	2.99160215373884	-0.01382933970340
H	9.00020418202042	1.81915359106414	1.24970258384114
H	8.49579310690660	3.44540321922440	1.69575877444143
H	4.99255900056655	0.99816880150173	-2.30023096220208
H	3.44349180057273	1.61139961680316	-1.71090990973118
H	3.88725957476638	-0.06369144944257	-1.41376641542664
C	5.07293138661267	-5.24542927245734	5.88572647324000
C	3.82784232867737	-4.42273635322247	6.23112377882015
N	5.31048842246823	-5.21875702503317	4.45225821001525
H	4.95942211691221	-6.27644543875544	6.22882814027238
H	5.95682690350804	-4.83157618393276	6.38730290526402
S	3.73529838714070	-3.18837208461564	4.90941419963141
H	3.90840731328949	-3.93001499724211	7.19923644309841
H	2.92033050990352	-5.02815793379365	6.21010582309631
C	4.75289789103739	-4.22697156450513	3.88631370725239
S	4.97639501463140	-3.83895221558564	2.20299921789156

Au(IMes)Cl

Coordinates from ORCA-job

H	6.80634604880788	2.10716850136532	-1.09586984630956
C	6.15989865027997	2.12390901503212	-0.22384171642987
Cl	4.70644264610742	-3.47014408776745	1.98556437842777
C	4.37503120485235	1.06287502907177	-1.63918365872633
C	4.86619073463427	1.62565833658130	-0.34139890404944
Au	3.43423852033526	-1.63454103740819	1.44675462777431
H	-1.18876440373969	-3.84594408915124	-0.61076221109309
C	-0.96727109984717	-3.16010079946328	0.20087947689363
C	8.04785876227109	3.14765702117258	1.08198793716661
C	6.64184806470375	2.64091975507589	0.97309015490890
C	-1.50880349586143	-3.39430966515906	1.45902050896321
C	-2.37717927622075	-4.58790609351112	1.71691137503330
C	-0.14232585049052	-2.06827178262603	-0.05313732003726
C	0.43982127966802	-1.84641017231117	-1.41469491293556
C	-1.21732031272175	-2.49915861533657	2.48472061273582
C	0.12894312544372	-1.20395567053207	1.00475088291125
C	2.31363211976852	-0.07749736370765	0.95436428129022
C	4.05330421503746	1.66237581769356	0.78981048579742
C	-0.40199125904310	-1.39402959961936	2.28210140271340
C	5.79379786803260	2.66119325281475	2.07640464946681
H	-1.63496703032771	-2.66397254486400	3.47338800298285
N	0.97636695711437	-0.07193199718299	0.78050797328420
N	2.70680024662923	1.18533907087126	0.69381489280578
C	4.49269855839264	2.17879744309032	2.00979927823484
C	-0.10337405728465	-0.44498033947755	3.40097488992173
C	0.53580255632504	1.18089056190640	0.41180532824707
C	1.62614533502228	1.97344038094743	0.35737405491261
H	6.15070857254586	3.06921030861128	3.01702945785533
H	-0.50434362568546	1.38917262272049	0.22590484279410
H	1.73454523530418	3.01692561234938	0.11378845591286

C	3.59767172187446	2.21675307718887	3.20972403577176
H	0.97326042646553	-0.34929336534956	3.56426947878648
H	-0.56233829819249	-0.79141621901094	4.32672688438879
H	-0.48504551880690	0.55679939569448	3.18567293192756
H	-2.61684011912588	-5.11076749173649	0.79039625947894
H	-3.31206616789859	-4.29742186560861	2.20256847054324
H	-1.87539087844308	-5.29491131302822	2.38404794242768
H	0.07381205509648	-2.59913416119638	-2.11274209956056
H	1.53181581215419	-1.90490109218544	-1.38895707987393
H	0.18077429537572	-0.85931152031044	-1.80602196235983
H	2.70089009620151	2.81332537904833	3.02125644940471
H	4.12165902811349	2.64913347161213	4.06192307657641
H	3.26176781961420	1.21349179301604	3.48537170917340
H	8.46362733430860	3.37716658127720	0.09985212666235
H	8.69325805775934	2.39675324223139	1.54844775788151
H	8.09786582483947	4.04559334929719	1.70113140902132
H	5.12943908327849	1.18317312217958	-2.41649358770725
H	3.45742665452226	1.55536214046956	-1.97080661020343
H	4.14972248280931	-0.00344339477480	-1.54299657379215

Au(IMes)Cys

Coordinates from ORCA-job

H	5.26266613998771	0.89221836280298	-2.52017724495390
H	4.26221680786911	-0.05286969966485	-1.40603410775788
C	4.56503055212494	0.95988263683614	-1.68556026861988
H	7.02934489654126	2.05566841200429	-1.56396648721170
H	2.09748795633549	-1.84776666248975	-0.87270412939873
H	0.71373919161371	-2.78423813486614	-1.45708226896465
C	1.00705643854346	-1.92602850556062	-0.85267156539591
H	3.66732485610694	1.47851966304618	-2.03274479276423
Au	4.14762883364737	-1.29040428848074	1.93543008708123
H	-0.46249761724324	-3.94416899819658	0.17195979237106
C	6.48869416195948	2.18363838725840	-0.63128420238520
C	5.19770048281599	1.67362977055853	-0.53141977473509
C	-0.25168716682989	-3.18428052824656	0.91833609086684
C	0.49927624639042	-2.07484614560890	0.54811254096140
H	8.88514035910651	3.22710846651141	-0.70815600014735
H	-1.24636643003199	-5.42600942983883	2.02735724757171
H	0.61230148012020	-1.02341872494927	-1.32683299591036
H	-1.51195752299976	-4.74884577608932	3.64512368592890
C	-1.56629623976539	-4.53804495193969	2.57590763272402
C	2.88040579302064	0.11007625157142	1.25191334055061
C	-0.73391530102913	-3.34604139315299	2.21329928015462
C	8.49359066393670	3.38247782846642	0.29774050442101
C	7.09951041750109	2.84727901752266	0.42548399833887
C	0.76565539716086	-1.11537684977732	1.52557230262563
C	4.52241225064550	1.84890598160067	0.67443077715711
H	9.16703623921337	2.89049770773819	1.00510932739167
N	1.53638560605741	0.03606568500652	1.16649154173177
N	3.18961939219989	1.34090654130718	0.79444691968284
C	-0.44510932226049	-2.36495970265146	3.15540108885260

C	0.30331015096789	-1.23685113849026	2.83517622135312
C	6.38668441326043	3.00231530576264	1.61136218638307
C	5.09508074778233	2.51320485977808	1.76044303448252
H	-2.61780698496389	-4.36303149243087	2.32731335878370
C	2.05220903707236	2.03013543407005	0.42836986156498
H	8.52457176621354	4.45293982773125	0.51721116854849
C	1.00937606306132	1.20738562684302	0.66307873304336
H	-0.80722398182365	-2.47886319803112	4.17260960074891
H	1.68752592812220	-0.09829621512776	4.01992951632989
C	0.60892175104827	-0.19966369200705	3.87076359910809
H	6.84729741354278	3.52006800194964	2.44739987376864
H	-0.04940059238358	1.34419673804158	0.52097446600601
H	2.09125219728811	3.03325959307310	0.03809880689896
C	4.34833650531913	2.69329197632755	3.04543187532051
H	4.07262777867746	1.72876463247176	3.48072274936562
H	0.15499075851693	-0.46822077482422	4.82452225347944
H	0.23216678170490	0.78360777123026	3.57695277689356
H	3.42139570559964	3.25287093506485	2.89341734574488
H	4.95906723414033	3.23419907314629	3.76811929768310
O	5.23963871223425	-2.55511532089413	6.33198742586769
N	3.02975892618403	-3.05282588276351	4.80528167601709
H	2.31353562793826	-3.33444377149784	4.14408378345977
H	3.35614260069887	-2.14004269406253	4.49857840789829
C	5.19278237539793	-3.60065232219680	5.73447622561226
C	4.11968990588160	-3.99962764536010	4.74661299494671
H	3.74164463753678	-4.97359182719072	5.08221595486705
H	6.78733340390579	-4.24156907809300	6.49740294736523
O	6.11391926298037	-4.55754798913415	5.87506750201503
C	4.74043274650466	-4.23809952603964	3.36009198148164
S	5.67622824074007	-2.83229938609635	2.67327158865234
H	3.94150413484127	-4.53036392382452	2.67728540952537
H	5.44633218926965	-5.06860881814300	3.42178505661663

Au(IMes)CysH$^+$

Coordinates from ORCA-job

H	5.28178910770515	0.97110549228505	-2.37745373092519
H	4.14913711721272	-0.01845181035290	-1.44446849300919
C	4.55156319776608	0.99047493362331	-1.56868623134301
H	7.08105330316289	1.86516091849913	-1.21370829779468
H	1.76664342881896	-1.80650333243909	-1.13553190315739
H	0.30950872285413	-2.55536655832284	-1.80461529013487
C	0.67337017503547	-1.78957602065455	-1.11992260801576
H	3.72092781394426	1.63170614236904	-1.87622263994786
Au	3.79958495819170	-1.68918093105852	1.73955164911928
H	-0.95263388637468	-3.77686547789888	-0.28903160734681
C	6.51262354406912	1.90164531302491	-0.28961106231758
C	5.18553348465452	1.48340300079521	-0.30501726663023
C	-0.68911662193002	-3.10933413172713	0.52570545616016
C	0.14545566725153	-2.03026304232539	0.26048422076750
H	8.94703011448045	2.87085778282969	-0.14727991075173
H	-1.83983635415526	-5.37161401111353	1.43241409432046
H	0.36233774986118	-0.81370761792208	-1.50208626948410
H	-2.08822419765328	-4.81534224453797	3.09847341331709
C	-2.10796505907653	-4.51265584461877	2.05044107492070
C	2.68096073481367	-0.11241833292158	1.20893378881582
C	-1.18900403027025	-3.35595507286327	1.80070318326591
C	8.56052171407395	2.78583557806806	0.86902201751656
C	7.12401190632207	2.36035395126444	0.87090394762957
C	0.47639290072014	-1.19188288507985	1.32527076963179
C	4.47641071707599	1.54022088525029	0.89282048181912
H	9.17948625401012	2.06020642320618	1.40507204128713
N	1.34181992719212	-0.07605880966887	1.08212593126558
N	3.10470595124944	1.12635409172329	0.89994991490505
C	-0.83456803419239	-2.49175918213675	2.83070894595534
C	-0.00343084606580	-1.39731296122187	2.61796652954383

C	6.37421927846975	2.40577417210106	2.04319837348822
C	5.04589049151137	2.00216655255027	2.08052058959667
H	-3.13996615100290	-4.24400782672216	1.80374895533321
C	2.03766275659578	1.93624152430765	0.57845368110486
H	8.68716361969068	3.74816998880764	1.37034829363350
C	0.92614953613622	1.17832854844093	0.69385570333665
H	-1.21262373963930	-2.67158454343058	3.83237127684739
H	1.44360077190177	-0.49878697568421	3.93066520018574
C	0.36591422534167	-0.48357731896874	3.74510682388461
H	6.83384091276599	2.76939086163727	2.95725820611366
H	-0.11349429967479	1.41286412864648	0.53877634430836
H	2.16784873527431	2.96881296965561	0.30134334167982
C	4.26156408726402	2.05942275944540	3.35461748842860
H	3.97745067430836	1.05755426900720	3.68872966274314
H	-0.14215365361692	-0.78473126996954	4.66093292582636
H	0.09380159106595	0.55205922845903	3.52591317129547
H	3.33811484111646	2.63078332636462	3.23150435123409
H	4.85164828259231	2.52545873770458	4.14341211675100
O	6.34890428310473	-1.02868335097940	4.74202248193109
N	3.89601645436855	-2.14005082468403	5.15724060702433
H	3.03098154258171	-2.66712928585979	5.13196991285507
H	3.87119834520066	-1.46789677841416	4.39666972453905
C	6.30154239861229	-2.21629539283586	4.93122807348415
C	5.02392148296710	-3.02351624796295	5.03305816897164
H	5.10808782980141	-3.61164163389013	5.95572431849431
H	8.17102879312226	-2.43723232205525	4.94718554364733
O	7.37918690652282	-2.98853956101532	5.05059515827200
C	4.96191971320660	-4.08970892858928	3.92793598144993
S	5.07919696634265	-3.56241113345309	2.19231106869750
H	4.00297024351033	-4.60710379815370	3.98496884316347
H	5.75508451128096	-4.82799670426254	4.05138350162933
H	6.32038910852923	-3.03888941627168	2.21680796066666

Cys

Coordinates from ORCA-job

H	2.38280854003274	-3.01304527333956	9.28329655838857
S	0.02305105362564	-1.27409286813238	10.16466566457999
N	1.86965275643164	-3.72764601036059	8.77869276484486
O	2.19757768278023	-1.56588948673243	7.15476753737866
C	1.09166708316558	-2.03499312459956	7.23994771891361
H	1.59655429276875	-4.43454981172211	9.45166437104517
O	0.05748505414679	-1.60438378861212	6.51650375050987
H	0.35751761531830	-0.87077021523993	5.95737758448287
C	0.69095215888551	-3.16193012766116	8.16689673628875
C	-0.40889633163697	-2.69798078532509	9.13267076405441
H	-0.69329160328782	-3.53446977743066	9.77213340830249
H	-1.29398428672964	-2.38502206354476	8.57682495185467
H	0.22656387853957	-3.92884609201476	7.53406236188029
H	0.94615210595967	-1.87867057528488	10.93074582747574

Cys$^-$

Coordinates from ORCA-job

H	2.21607466943926	-3.03625761505271	9.47525790075249
S	0.31777555669298	-1.33202376599817	10.26705828173513
N	1.92675326572225	-3.69369839234660	8.75127655100208
O	2.17371183701684	-1.56040563742335	7.02298115931956
C	1.07939101642849	-2.04041400724547	7.18885768868194
H	1.67170235266664	-4.54406801418905	9.24288497616536
O	0.01037541393192	-1.63528318771461	6.49146016247350
H	0.29241685650096	-0.92192034943142	5.89894903286233
C	0.72360808076115	-3.14666441904984	8.15645680535760
C	-0.31613924000593	-2.64593301273206	9.17597148924884
H	-0.61794892834658	-3.51693801506599	9.76676386133163
H	-1.20207683418361	-2.30159761938503	8.63684490913714
H	0.24885595337561	-3.93049596436569	7.55253718193230

TS1^{H2O-PT}

Coordinates from ORCA-job

H	1.60074962722122	-2.22367519514119	9.79858835723386
S	-0.61456875356714	-2.80688504368226	11.18983570584955
N	1.64539363339261	-2.71643344444517	8.90952428826322
O	-0.14130556135622	-0.64619299515593	8.33743544328787
C	-0.38016955811863	-1.77704745437318	7.96915617426938
H	2.12374904311398	-3.59025386972709	9.10167310921932
O	-1.32733767426258	-2.05545393170982	7.08188242543405
H	-1.76944593398326	-1.22988494110279	6.82562588801081
C	0.29366518174088	-3.01908419264825	8.49490488658197
C	-0.61153992698095	-3.65113700666013	9.58048211649290
H	-0.23171557803626	-4.65804498759155	9.76526550793765
H	-1.63045141521784	-3.75194647019352	9.20060968138128
H	0.33131935552114	-3.73254329808114	7.66694112390592
O	-1.73406648643453	-0.31569637579163	10.56359448984653
H	-1.30988665953056	-0.08559657714548	9.71381630807727
H	-2.69005434386546	-0.31179733870255	10.41374806671552
H	-1.29784494963633	-1.44638687784789	10.83298642749151

AuIMes2tu – TS2^{H2OPT}

Coordinates from ORCA-job

H	6.24304799866947	-2.15034831147703	-0.99122974890848
C	5.90561813484145	-1.47004829678244	-0.21519587519865
C	3.55985898009530	-1.87446771404677	-1.01438482312782
C	4.54134578043479	-1.22357596662856	-0.08968571291662
Au	2.25848060520558	-2.52321865338179	2.83616771291519
H	-3.06327626609033	-2.83633735799663	1.54941483974683
C	-2.47534970795585	-2.09348022471028	2.08020978857589
C	8.30544267017390	-1.13836981086804	0.45797309521892
C	6.83979956544589	-0.86962373253799	0.61991842984421
C	-2.92975363612511	-1.60685124593453	3.30188482085935
C	-4.19902483632695	-2.12406894586912	3.90761645740776
C	-1.28550703635791	-1.65316547909276	1.51229838515209
C	-0.81292481008103	-2.20209821171580	0.20194879732997
C	-2.16749551899815	-0.64569905059862	3.95670768853991
C	-0.54622193463658	-0.69543046201100	2.20749449790849
C	1.87696861165870	-0.82904157374766	1.80607110616141
C	4.12630053230626	-0.35356912560094	0.91697040730987
C	-0.97096989103650	-0.17286734159771	3.42829368859700
C	6.38353900152737	0.00326319362365	1.60401109700749
H	-2.51110661638804	-0.24814708848965	4.90695334838500
N	0.67669867112757	-0.22451825261271	1.63876925221938
N	2.73101284013842	-0.08319035880156	1.06459326528629
C	5.03207792956681	0.27849655143558	1.77117822717520
C	-0.16787650620382	0.86333767330656	4.15188852293883
C	0.78071098107213	0.87839687956890	0.81269132537265
C	2.07480366452937	0.96681915690491	0.45020327772839
H	7.09892201176488	0.48422899770421	2.26458322269937
H	-0.07048330448316	1.48854632342458	0.56118022114306
H	2.58870564666550	1.66994272213962	-0.18357611547691
C	4.56648661962594	1.22134263891648	2.83746897732440

H	0.85066922391945	0.51292740791964	4.33872375276882
H	-0.63085179378130	1.10556108117765	5.10853385419518
H	-0.08734885491615	1.78480244836494	3.56865435412829
H	-4.93036686678704	-2.37799624719636	3.13800799194345
H	-4.64308921165264	-1.39189849077725	4.58403380018161
H	-4.00741128376279	-3.03298118700865	4.48691831935800
H	-1.57537413508144	-2.84369771240978	-0.24001852065400
H	0.09810114107069	-2.79369430647971	0.33493544665554
H	-0.57769767122063	-1.40510560072977	-0.50770203896270
H	4.01748426370347	2.06567264162334	2.41204607001949
H	5.41607278117970	1.61437478665946	3.39630219271569
H	3.89429656451777	0.72158224781284	3.54001912548301
H	8.48235458008698	-1.95741961710646	-0.24029459101013
H	8.82396129886454	-0.25319667895578	0.07767114458579
H	8.76718082183547	-1.39402132029455	1.41477813746390
H	4.08053541686068	-2.47040559970630	-1.76406166351614
H	2.94522416507083	-1.13237306417288	-1.53074689267661
H	2.87718677347888	-2.53159169147188	-0.46838470476146
N	3.34257513788011	-0.90174742770159	5.91365717842263
C	3.74763153542975	-0.06795416484566	6.89887192876083
C	3.59633927270767	-2.18244090162158	6.07573582362586
N	4.23894837815735	-2.62423610724927	7.19445197777230
C	4.68144683723988	-1.82679505294142	8.24061386995434
C	4.39105810457504	-0.44300645091513	8.03928463486220
O	5.25661301140434	-2.33870260561979	9.20056056660227
S	3.15394585612301	-3.41404524355889	4.94681986206354
H	3.52216103388989	0.98243838951439	6.73206842474997
H	4.69023485263031	0.27401167778566	8.79137467141387
H	4.42011025991804	-3.61621085875361	7.27793370408978
H	5.10693998823381	-4.83606383471585	2.94879688628994
H	2.97277946981751	-5.89882930029644	3.66183577475407
N	4.95029756168541	-5.03256444271568	1.96505521592528

H	4.43068757055040	-4.23156324822002	1.61257772610642
C	2.75205521977930	-6.09514546831077	2.61025208811027
C	4.09683119067846	-6.19841754510555	1.86994050566467
H	4.63438425925547	-7.05244909059246	2.30226890478587
S	1.64461668286244	-4.81348959000237	1.94736209685578
H	2.23970163473285	-7.05798319187811	2.55344425365912
C	3.86819249605309	-6.54463230145223	0.41592480040809
O	4.20990230941371	-5.88101002913771	-0.53137276025967
O	3.22883110016062	-7.71085351714946	0.27998252515912
H	3.09785884326192	-7.87267572428281	-0.66690461491966

AuIMesSpym – TS2^{H2OPT}

Coordinates from ORCA-job

H	7.15232202405751	1.39408894442402	0.67186673774300
C	6.25444827069207	1.79616746011683	1.13142484114045
C	4.96206077091388	-0.02428463655084	-0.01955654603480
C	5.03637082478152	1.17691225100411	0.87173802893000
Au	2.92300254313317	-0.81725330596140	3.56528187512123
H	-0.76439203255813	-4.41595608131315	1.35867105133788
C	-0.84098310860290	-3.39741359962235	1.72732318777707
C	7.66987283216203	3.57750730298141	2.19995197529117
C	6.35047478878666	2.90822220938398	1.96064032237110
C	-1.87003171883393	-3.06297276895544	2.60185721121294
C	-2.84917054899204	-4.09962235285823	3.06272653140604
C	0.09432027391914	-2.46125843306554	1.30184572534757
C	1.18407222971660	-2.84495507694290	0.34953728453864
C	-1.95546428357500	-1.75015897744502	3.05196551272774
C	-0.02164472378806	-1.15764849052915	1.78556565411374
C	2.09917441361125	0.08861626691536	1.94767814473075
C	3.89557370207435	1.70457281316780	1.47580606431502
C	-1.04069800026593	-0.77865030088298	2.65872842374616
C	5.18811015980036	3.40117744794289	2.54484832553389
H	-2.75706039407951	-1.46847955707494	3.72801109763721
N	0.91327435633387	-0.16997369921679	1.34767954134345
N	2.63008148591702	1.09514504889798	1.21420779324281
C	3.94730131517008	2.81802575036242	2.31521429423710
C	-1.15356373335846	0.62765466094353	3.16153268603641
C	0.71069289505854	0.65821050812822	0.25940098063315
C	1.79243887823479	1.45590111805861	0.17567366319368
H	5.24545152797413	4.26498713761645	3.20043672933184
H	-0.17886255151175	0.60181664945402	-0.34532617471526
H	2.04540604858163	2.23961990236203	-0.51855005872233
C	2.71134814218227	3.36690556549898	2.95843612722329

H	-0.25756540014041	0.92143485262923	3.71560433913845
H	-2.01411129756725	0.72815905436321	3.82297298343102
H	-1.26859525242326	1.33876014491575	2.33916641212664
H	-3.05892070013917	-4.82493321976755	2.27418541191566
H	-3.78948227183738	-3.64429633699813	3.37754683216644
H	-2.44901765451845	-4.65489399804263	3.91695847812538
H	1.11801878562603	-3.90427045644684	0.10116946840021
H	2.17021037038002	-2.65072672203395	0.77958009127366
H	1.12108677372165	-2.27112530993685	-0.57920416399132
H	1.95822433289396	3.63487871903940	2.21290412552356
H	2.94757447642232	4.25680004850027	3.54191631592557
H	2.25413912204627	2.63016257363989	3.62516874563716
H	8.49850121053425	2.88112143022998	2.06165126327666
H	7.72616337756983	3.99212241900247	3.20820957566787
H	7.81542347165130	4.40557801633035	1.49896582048104
H	5.94549401389768	-0.25810875172414	-0.42748173501353
H	4.27378925268524	0.13651465168436	-0.85343205122062
H	4.60056351980405	-0.89831160946660	0.52976459803593
N	6.01585847726975	-1.76467432117149	5.39512460370571
C	6.96378171068681	-1.83650700607304	6.32183198471270
C	5.53222656449402	-2.92313835753218	4.92110915397364
N	5.96370553543376	-4.13673645425496	5.30885210624907
C	6.91587702238999	-4.17649121637439	6.23018276807440
C	7.46456586738479	-3.03651133936756	6.79385943630827
H	7.25386008055175	-5.16507915351283	6.53202255345115
S	4.29613273155217	-2.89875932614978	3.68944066773918
H	7.34050234745557	-0.88880856989207	6.69912427540520
H	8.23755067902937	-3.08150422890350	7.54907104021548
S	2.49011185583073	-0.07329255068713	5.99819962251689
H	2.42358973917012	-3.77923623180894	4.99329007286254
O	1.61038847957324	-3.47958759542989	5.45333153256284
O	2.96835681192864	-3.40073505969646	7.22544107796361

C	1.95202685232584	-3.05794711897049	6.67214803047927
H	0.41458870325618	-1.14618978628236	5.36112509281125
C	0.87096782959014	-0.85771291567854	6.30880896137449
C	0.94102426412478	-2.08639889683511	7.23360879615076
H	2.15123806704369	-1.31806922264877	8.64777123518725
H	0.20729188003586	-0.13205727405614	6.78804629479592
N	1.25494244057110	-1.79637212900711	8.61798686489097
H	-0.03874999925167	-2.57965069685102	7.19667478536147
H	0.57079954140769	-1.13479981157348	8.96922550151203

AuIMesSbtz – TS2^{H2OPT}

Coordinates from ORCA-job

H	6.99308733392931	1.41723287593534	-1.35780193958599
C	6.54445565179219	1.30776260471344	-0.37509657319052
C	4.39457837172700	0.70538108189082	-1.52555647518330
C	5.20624966602804	0.93711915869577	-0.28900803058970
Au	4.03235547744712	-2.51806677087743	1.25703437454010
H	-0.92670844236767	-4.34188469850039	-0.20618282958822
C	-0.62512176621320	-3.72586746457793	0.63575797169988
C	8.75228832640954	1.94974961247799	0.64060663744173
C	7.31716993759751	1.53410462545638	0.75809389031114
C	-1.11943989184455	-4.01273108693153	1.90488452049387
C	-2.02674986346626	-5.18494593483230	2.12443197701829
C	0.24128737169606	-2.66394476925500	0.40852308281095
C	0.73526951320600	-2.36125836201424	-0.97121461613666
C	-0.74149015057147	-3.19322174872176	2.96133866446914
C	0.61925681121824	-1.88286318248810	1.50244375299292
C	2.86152094536438	-0.88261746606117	1.24084043463809
C	4.65116889006101	0.78817613159253	0.98112819357809
C	0.12295808549722	-2.11698436400061	2.78440912742281
C	6.72071270915131	1.38621472535262	2.00654145487408
H	-1.13001119531588	-3.39137558913494	3.95570156144756
N	1.51036638876121	-0.78414296994082	1.30090124431094
N	3.28110480978880	0.39812239439837	1.09431318180610
C	5.38751335318140	1.01835951518590	2.14429674038177
C	0.47583623281748	-1.22889990714179	3.93735554363153
C	1.10026632780643	0.53167525007394	1.19349308202906
C	2.21487883751337	1.27559388559018	1.06221981899045
H	7.30925617323856	1.55990512252450	2.90251014026573
H	0.06029369173490	0.81052856152495	1.22152844683272
H	2.35057266402314	2.33801417071304	0.94891132715259
C	4.76730006337088	0.87635382454031	3.49948439117848

H	1.54190060757234	-0.99649289224605	3.95921068829652
H	0.20448442657956	-1.70290225964500	4.88096549969731
H	-0.06029297181963	-0.27700194258624	3.87444365105920
H	-2.58299798050811	-5.08573891037190	3.05770737887167
H	-1.45127200407783	-6.11436347429399	2.17804235832193
H	-2.73865003107542	-5.29281483817637	1.30342510498464
H	0.32084018198315	-3.06856244206102	-1.68975458283001
H	1.82545582991736	-2.41706091162696	-1.01796938243681
H	0.45244327057118	-1.35108179857107	-1.28039086416972
H	3.93319148402266	1.57203840426638	3.62767876602266
H	5.50221943862597	1.07735231212042	4.27900410451431
H	4.36844183799565	-0.13020060124600	3.65102886279803
H	8.85132074374942	3.03446554535019	0.74848704220744
H	9.16515882983595	1.67514107579767	-0.33128266264621
H	9.36210028585949	1.49023157968206	1.42107755866592
H	5.01823933479726	0.79819017366341	-2.41476005589840
H	3.57759545615636	1.42799009705679	-1.60574206576417
H	3.94232060625553	-0.28916510120467	-1.52275069974758
C	5.00757839789003	-4.51513576475312	6.44963260428265
C	3.85817506462696	-3.69875656557182	6.45428365560242
N	5.61688361213483	-4.67390260018060	5.22768862671605
S	3.57611718310705	-3.13436683730140	4.84223199094484
C	4.99278401406335	-4.01982140298375	4.28913321516257
S	5.45022603762423	-4.01338554718470	2.63056783875050
C	5.43579481196938	-5.09180793110863	7.64646941648981
C	4.72087129046119	-4.84974960637165	8.80657739307387
C	3.58377284416899	-4.03895005621246	8.79599002912142
C	3.14192948970055	-3.45529417254402	7.61838866650887
H	3.03868520235322	-3.86350884983568	9.71643823386904
H	2.25924023428146	-2.82615143015854	7.60877010041508
H	6.31826363448868	-5.72193640423824	7.65222985322640
H	5.04817197054163	-5.29698029950293	9.73857110443766

H	7.93944065330938	-4.33382907157143	-3.65800022677942
O	7.08471744258430	-3.87614025075561	-3.65219522958581
H	5.40070861062376	-2.38396964522623	-2.36866245423380
C	6.12586913319162	-4.79019782310791	-3.46824086880661
O	6.36447334809666	-5.96730860287651	-3.36262896081685
C	4.66015669062985	-3.17548782060288	-2.23863432335597
S	4.89853921604781	-3.99636130822482	-0.62838628107456
C	4.75356613042234	-4.15584531412674	-3.41965674459594
H	4.66466508437777	-3.56130250801634	-4.33744363364977
H	3.67165055974852	-2.71264290092247	-2.28456112031911
N	3.73458468434786	-5.18503785374018	-3.44877414873445
H	3.74883074167971	-5.67006349728983	-2.55536208657354
H	2.82848224750159	-4.73186017768489	-3.50424544806749

AuIMesStzn – TS2^{H2OPT}

Coordinates from ORCA-job

H	6.82950474660003	1.66124536248260	-1.27042457296526
C	6.36441338738874	1.51632945518998	-0.29997593265474
C	4.25786852426229	0.88008570003041	-1.50892912592032
C	5.03754871822186	1.10313002814388	-0.25045746502147
Au	3.84338299549050	-2.42940494169564	1.15652561014412
H	-0.98729930104684	-4.23851417059315	-0.55942867399994
C	-0.75130993997792	-3.63384354896833	0.31117034566062
C	8.54296861689463	2.15900913475447	0.77558086606965
C	7.10329223744986	1.75070037019020	0.85454225171102
C	-1.35104056840728	-3.92924730177735	1.53160398863923
C	-2.28391794781758	-5.09445419570202	1.66359628302324
C	0.13824859486125	-2.57618718601310	0.16626717292191
C	0.75155844925920	-2.27293916016616	-1.16486920695521
C	-1.05273131955233	-3.12480647513768	2.62459876147803
C	0.42942074381202	-1.80694898802351	1.29487757860135
C	2.68014465999684	-0.78639316478897	1.16276163723522
C	4.45671396869559	0.91875750797786	1.00426093213612
C	-0.16894552651455	-2.05427158467050	2.53039233234205
C	6.47898999139171	1.57774896357726	2.08543147280824
H	-1.52142301966805	-3.33040184208230	3.58225474850291
N	1.32582949836238	-0.70049639917162	1.17568656639558
N	3.09143121579325	0.50332102897999	1.07639010539519
C	5.15528758882726	1.16443302809435	2.18668485726985
C	0.10491792979283	-1.19070282838548	3.72283507013597
C	0.90713126264052	0.61468981009253	1.09647660844396
C	2.01812339926104	1.37191546484799	1.03366165466518
H	7.03521811560595	1.77372499521358	2.99728085566736
H	-0.13586303741541	0.88333162209235	1.09242707520393
H	2.14765494166640	2.43863634633524	0.96067315844638
C	4.50515122221092	1.00040789261361	3.52495891305118

H	1.17371641377327	-1.00960599527557	3.84985518263666
H	-0.27551728244545	-1.66256164656064	4.62902902769870
H	-0.37988453114905	-0.21502382459535	3.62315124919057
H	-2.91487481520832	-5.19804478920269	0.77843710871977
H	-2.92599602373614	-4.99074616309713	2.53962206113998
H	-1.72308109450100	-6.02807339906492	1.77234008937720
H	0.32236509651957	-2.91149068978989	-1.93719784489646
H	1.83219637939026	-2.43736448344814	-1.13998113450746
H	0.59264654219078	-1.22956077907753	-1.44943203988397
H	3.62447131665109	1.64144302487781	3.62087699954044
H	5.20326640273842	1.25818296896260	4.32162142600777
H	4.17110439554209	-0.02855601572516	3.68202464316281
H	8.75538262879683	2.68495899150128	-0.15672083538962
H	9.19503811649595	1.28054538333215	0.81255936921834
H	8.81862052384709	2.80381989579014	1.61208608693372
H	4.90954560428642	0.95045479036129	-2.37993824565423
H	3.46283815452979	1.62352593239099	-1.61810887820833
H	3.77972564454303	-0.10180621845683	-1.51183209387481
C	4.90339341731221	-4.15806398550259	6.55628913178663
C	3.56498518308819	-3.41625788361998	6.53587465827722
N	5.23817261101247	-4.61850673072336	5.22089365089211
H	4.86599255627894	-5.00121525303329	7.25105735278206
H	5.70239846826952	-3.48915244132188	6.90361865723094
S	3.47499400166363	-2.73073452466511	4.86546929678710
H	3.51898590673055	-2.61840016891737	7.27665026336174
H	2.72237285616535	-4.09362608340793	6.68738628134602
C	4.65061716413715	-3.95262617294992	4.30385766927545
S	4.93376263479687	-4.15016667208557	2.60645969031691
S	4.99006031146751	-3.75269191369120	-0.65504478557157
H	8.18659479775097	-4.11034889594306	-3.51654012745778
O	7.33835032872577	-3.64281157227418	-3.56177524167558
O	6.57867726998127	-5.72306960357590	-3.29566189515772

C	6.36025873025902	-4.54404230032145	-3.42082604716061
H	5.60815758270073	-2.13378583428827	-2.35095422200036
C	4.84971483846916	-2.91471395872411	-2.26760571492845
C	4.99500857304113	-3.89302343274624	-3.44564942989993
H	3.92077864441270	-5.38588310908822	-2.63316887688679
H	3.87198886644898	-2.43788792439625	-2.36402079783524
N	3.96706188440215	-4.90975792898841	-3.53059512109147
H	4.96288257700247	-3.29547320281697	-4.36494965521611
H	3.07191717552932	-4.44585231327980	-3.64435077681844

AuIMesCl – TS2^{H2OPT}

Coordinates from ORCA-job

Au	3.28853417952083	-1.68857463735076	3.02386405687715
H	4.92594933435040	-1.31364778024246	-0.28110029003839
C	5.25939496837305	-0.34607733974088	-0.66767027648910
C	2.51024137746428	-0.53598225940421	1.59401090182988
H	0.28118700772790	0.20157670726654	3.49315165913616
H	6.19898277284808	-0.49861347486650	-1.19894379119950
C	5.44448232844293	0.63572162201730	0.44731511013299
H	2.43050475926260	-3.18361907593475	0.14708960520696
C	6.69976694750745	1.16527410539328	0.72627049860193
C	4.37506342411281	1.04136717027849	1.24577087359653
H	4.51016320670054	-0.00528628571800	-1.38656264770824
C	0.33537823037510	-1.66631386551174	1.36393585417352
C	-0.66174301158907	-0.00582557292425	2.97975131018566
H	2.95957064597855	1.57121265998047	3.69774331797022
C	-0.63139313368718	-1.34982767580975	2.32003977693334
H	7.54236112108787	0.85891067655608	0.11379308588533
N	1.29981822260929	-0.67381692905136	1.00635933966631
N	3.07238814278911	0.52234708159988	0.96660759582743
C	0.38308963802857	-2.91071935187990	0.73894545826540
H	-1.46965754321653	0.04220668400314	3.70986680093029
C	-1.56144086272780	-2.32720057270239	2.65010007563414
H	-2.67741736416278	-4.69331662316236	3.50922157197280
C	1.42697403942467	-3.23014702309448	-0.28573974487816
C	4.52869984147721	1.96080870656405	2.28350156982983
C	-0.56926608539333	-3.85713914095278	1.10519513677282
C	-1.54657111179532	-3.58588163929174	2.05528722397783
C	6.90113374410868	2.07028476749956	1.76274804715127
H	8.76606721197731	1.96776076584511	2.81373790586771
H	-2.31532619022567	-2.09798353566605	3.39733677565169
C	3.36402001200608	2.40318535268227	3.11424546547410

C	5.80411034205819	2.45949417866060	2.52445918812031
C	-2.57093332062898	-4.61548675455196	2.42470404154186
H	-0.54177995667827	-4.83440913604227	0.63303584402426
H	1.27422228578613	-4.23250755036295	-0.68595782385293
C	8.27100556172584	2.59617552921152	2.06653167664837
H	-0.81190415284565	0.79515808369869	2.25080708031169
H	3.66553330669286	3.19129064453445	3.80438989137594
H	8.90066353818883	2.60193709426954	1.17512376044204
C	1.10835057286601	0.28370310429608	0.02986345609334
H	5.93957418509011	3.17825823283902	3.32701621437953
C	2.22431306626209	1.03776106007231	0.00569995900789
H	1.40126960657166	-2.52081047399744	-1.11702103122083
H	-2.30463571929853	-5.59740545712518	2.03147043171807
H	2.54925481840755	2.78264493577826	2.49222236067988
H	-3.55318308895525	-4.34822991815358	2.02344416806321
H	8.22658927509653	3.60931009851072	2.47037414399429
H	0.20172103777992	0.33776142098413	-0.54905110257874
H	2.49473520168805	1.88615389697394	-0.60020393035291
H	6.86178381699242	-1.59701174135972	2.89445133932656
S	5.11230851954199	-3.45339122899339	1.89525437955739
N	7.52406838763069	-2.01423078232302	3.54585821491225
O	8.73122944288037	-2.88150797940696	1.22583429093136
C	8.33528380409167	-3.70823480212700	2.01008476259481
H	7.12237075548706	-1.87698544546605	4.46764616047763
O	8.55677390278093	-5.01693066816147	1.83924438484684
H	9.04339421257671	-5.13258498013367	1.00888562955226
C	7.55749743982350	-3.43837043700373	3.27837315465835
C	6.17807175360128	-4.11779882201858	3.20821560884668
H	5.69069807986230	-3.96422291923074	4.17587210885779
H	6.31574994562577	-5.19270694299862	3.07114153967625
H	8.12095515216188	-3.92440398936503	4.08483336350853
Cl	3.57995237375637	-2.54756176738828	5.24586446661772

AuIMes2tu – TS1$^{SPT/NPT}$

Coordinates from ORCA-job

H	5.99902055401256	0.63019758250432	-2.22703336566082
C	5.74894801556334	0.76857270705747	-1.17913854171331
C	3.41398297873024	0.02925411913385	-1.73667045034963
C	4.44648550020676	0.50153435153951	-0.76166970286862
Au	3.04553536294173	-2.56761999100892	1.44298329534311
H	-2.23041037053169	-3.56073922362026	0.99314311390815
C	-1.64592157519090	-2.99833309726711	1.71495146250408
C	8.10701012048420	1.54404874476293	-0.78011444680765
C	6.72509790861422	1.22666167082751	-0.29761655430455
C	-1.84686641451584	-3.21465490481028	3.07449419973334
C	-2.87897391157154	-4.19136887849033	3.55035064641958
C	-0.71494881662851	-2.07771916153963	1.24781143421401
C	-0.52616739790511	-1.85507050855816	-0.22063203625302
C	-1.08645751253748	-2.48405468313793	3.98103300209115
C	0.03123573082136	-1.37280409769760	2.19268527618089
C	2.26887196600342	-0.70605538324241	1.40374064288773
C	4.14494950953984	0.69523251156676	0.58561560672975
C	-0.14141350649586	-1.55277733507477	3.56464046149347
C	6.37637541685562	1.41299995298672	1.03563211955374
H	-1.22783473126089	-2.64192648094462	5.04585036104525
N	0.99292887735869	-0.41510022542821	1.73892312103993
N	2.80400941972601	0.47734181471216	1.03374476501049
C	5.09122744533478	1.15954796506046	1.50077933475753
C	0.66175264003493	-0.77702979373359	4.56189928054643
C	0.73169460957342	0.92976205838390	1.57877933219662
C	1.87213462883984	1.49246733767918	1.13563608923479
H	7.12403540773038	1.76768960281096	1.73821629010549
H	-0.23468008455360	1.35381295212349	1.79384866904293
H	2.11072329152373	2.51161435932683	0.88194071922422
C	4.74231945255040	1.38061912419779	2.93884680892150

H	1.73365366157663	-0.95222168480464	4.43475713577028
H	0.38690819796063	-1.06474879274115	5.57676804152110
H	0.49677471367783	0.29871002287086	4.45701812194191
H	-2.97892649519807	-5.03043842304115	2.85941719912811
H	-3.85968230134867	-3.71060431003255	3.62330705211729
H	-2.63040869598916	-4.57983896336123	4.53929736910096
H	-1.20372869731796	-2.48886397350876	-0.79258910650746
H	0.49904520841628	-2.08078690635251	-0.52635445638579
H	-0.71884783257800	-0.81384547728660	-0.49324625741608
H	3.94587007544683	2.12200778399993	3.04472891435843
H	5.61317126128419	1.73635102699546	3.48948379276413
H	4.39241138670586	0.45684429855400	3.40788341103524
H	8.18479856560746	2.60285930330809	-1.04634114855664
H	8.36102280447368	0.96314166512287	-1.66814195969679
H	8.85077406415075	1.34678269884201	-0.00622718097855
H	3.83301520803999	-0.01946114123664	-2.74152125752128
H	2.55084664178802	0.69994538532701	-1.75601251820436
H	3.04255355838148	-0.96331105507242	-1.46797146514546
N	4.37635756678233	-1.60003825849312	4.94125413965606
C	4.80485301729218	-0.94964908218089	6.04647869021340
C	4.25515146580391	-2.90950268212405	5.02877922048941
N	4.55787598988068	-3.55416619190843	6.19238182581564
C	5.00459654433080	-2.94973337427605	7.35998588262616
C	5.12315066733055	-1.53224383571943	7.23582146094197
O	5.24946150074460	-3.63586921111602	8.35194648118953
S	3.72629274067570	-3.89451870992079	3.71985026987406
H	4.89189273071614	0.12886024496878	5.93872787704464
H	5.46289956765395	-0.95465493993486	8.08451027541678
H	4.45178857174125	-4.55991659503256	6.21755500688899
O	6.59895438412327	-2.43507529922153	1.35790104291876
S	3.35155881718689	-4.71687590068400	0.38585164188218
H	3.67559703553502	-5.54305596793850	1.39908571465964

H	5.81250355776867	-4.79904124527417	2.16731577253900
H	6.27246254136184	-1.42733538533191	-0.70725605830511
C	6.34869317269261	-3.00882804550614	0.32914559773392
O	6.19946300460405	-2.39097133235793	-0.84014102748075
N	6.43978212182080	-5.15302399605520	1.44947811461053
C	6.19568882246141	-4.50972185493872	0.18541655791797
C	4.89880701838575	-4.89497201483085	-0.54153731199357
H	6.29384356963064	-6.15238632322000	1.37032995215097
H	4.76326796000505	-4.30698499720285	-1.44920056539375
H	4.94739636856110	-5.94848640788832	-0.82143888232754
H	6.99002542257518	-4.80328314151326	-0.51491830062273

AuIMesSpym – TS1$^{SPT/NPT}$

Coordinates from ORCA-job

H	6.79225819092654	-1.56543736840596	-0.49636558620427
C	6.28188623042499	-0.91153536069979	0.20425223909444
C	4.10912253522383	-1.75134056334750	-0.73601371238449
C	4.89158868706285	-0.89290770080742	0.20812822948402
Au	2.76262267157953	-2.19696237252755	3.26959206116782
H	-2.69054932232199	-3.40042445925959	1.70025670023663
C	-2.15758733454030	-2.60410549356986	2.21129885359276
C	8.52576271585695	-0.18962655686050	1.07541235939352
C	7.02899532120234	-0.12013888625577	1.07017510446907
C	-2.60890947401549	-2.16393477036738	3.45247391882352
C	-3.82616651989743	-2.77089231885579	4.08088489792719
C	-1.03820881773434	-2.05323840118094	1.60103087917512
C	-0.58596928828861	-2.53434867508623	0.25707566883690
C	-1.91033906346963	-1.14411527018582	4.08656928917988
C	-0.35976534308696	-1.03890354529818	2.27899833376335
C	2.06949104220023	-0.81581543425526	1.93962892914977
C	4.25575955358506	-0.04997866117779	1.11826240569560
C	-0.78111641890109	-0.56165354108205	3.51857283660012
C	6.35313359007632	0.72027430691437	1.94874547070884
H	-2.25091614339542	-0.78563734345015	5.05308239520139
N	0.79738253769323	-0.45809400177207	1.66949141850329
N	2.82503172835462	-0.02268560244068	1.15154518534841
C	4.96475040782733	0.77447817352239	1.99164944197105
C	-0.05166834288501	0.53645389780302	4.22897020181656
C	0.75712123077143	0.54453936917304	0.72266794174156
C	2.03667375375264	0.81908062920480	0.39593147815757
H	6.91991047878495	1.35433460570943	2.62372243790573
H	-0.17098988026885	0.96632733648454	0.37519705880780
H	2.45694176029068	1.52978642986947	-0.29563660714858
C	4.26239396929421	1.68559328199707	2.94954375942387

H	0.95186500879297	0.21679892587058	4.52383633793685
H	-0.59360541807102	0.83147764903192	5.12749583208490
H	0.06606456002793	1.41715146554723	3.59250682524485
H	-4.72127539441924	-2.54428134503175	3.49440791783008
H	-3.97975088006105	-2.39170089977996	5.09185667304797
H	-3.74196982531444	-3.85946124579704	4.12974674609372
H	-1.13786724019603	-3.42884033326470	-0.03180672970996
H	0.48112207371666	-2.76880796527212	0.25098564938211
H	-0.75091625173637	-1.77343169093570	-0.51139570112989
H	3.61771889130208	2.39609623599918	2.42528937509035
H	4.98546545684529	2.24965767579722	3.53844885826046
H	3.62560261087895	1.12028065655161	3.63576925948482
H	8.91344923460955	-0.47569517058263	0.09623657226202
H	8.87285458487843	-0.93655513886227	1.79646035574835
H	8.96608918184410	0.76742308989997	1.36080148582968
H	4.77961319900903	-2.36985900426369	-1.33276219643177
H	3.50542369679602	-1.14591305158725	-1.41781178327009
H	3.42196079081905	-2.40907811336315	-0.19739379062973
N	3.51947527061885	-0.91933360757903	6.32527579198273
C	3.70099124802293	-0.14951254307264	7.39336522742053
C	3.98200063730559	-2.17088861633807	6.39857305013974
N	4.60777180315528	-2.69671088559190	7.46205930331967
C	4.77211417777014	-1.90676609082812	8.51466937810385
C	4.33071505564626	-0.59441150241806	8.54144349849015
H	5.27881887117456	-2.34361053267934	9.37149228009748
S	3.80254143557359	-3.27993238157966	5.05595365405940
H	3.32286271188906	0.86716734567669	7.32256265615778
H	4.46933456066123	0.04196023164623	9.40489938062309
O	5.01736299882672	-5.79854959232757	0.78935432373221
S	2.03941987733835	-4.41343034253183	2.50786377649557
H	2.39170080577619	-5.12371991730118	3.59159403670887
H	3.93566756531521	-6.81809207442632	2.83407973966590

H	4.15519189054730	-5.01284476918768	-1.19512826104219
C	3.89751395429254	-5.99241003255560	0.38950685705203
O	3.44618769982428	-5.52427723515913	-0.77482428212848
N	3.43007296751525	-7.46832246913563	2.24258347376337
C	2.83201693796046	-6.77278932682951	1.12665868021245
C	1.63796596767463	-5.86805526134857	1.45954724668201
H	2.70580494643071	-7.89489789776602	2.80895281432509
H	1.20434889987515	-5.45154528556855	0.54940512416770
H	0.87195082634945	-6.45931790233938	1.96406661919046
H	2.45211215463072	-7.51767175450778	0.41553035321649

AuIMesSbtz – TS1$^{SPT/NPT}$

Coordinates from ORCA-job

H	7.04181286783884	1.20325006846086	-1.07356682391073
C	6.56042785738074	1.14739264953427	-0.10203689591380
C	4.40458419983769	0.67378418206011	-1.30017043906824
C	5.19722770073497	0.87704714505851	-0.04661360397473
Au	3.67539501010570	-2.49099065272428	1.55199234067393
H	-1.44563203684623	-3.59079233829737	-0.55011488406535
C	-1.06774680691014	-3.14009573663690	0.36254839651825
C	8.77968840606736	1.65490390612894	0.96314930199261
C	7.31602840644905	1.34501909686574	1.04797201621122
C	-1.54769603933519	-3.57985055257828	1.59215196288614
C	-2.54820588371085	-4.69250221024483	1.67043052350851
C	-0.11650621431680	-2.13131503038493	0.26434239330077
C	0.36121131272367	-1.65569892421021	-1.07251942141362
C	-1.06513537593654	-2.97373201505557	2.74682160359792
C	0.35712736165592	-1.56814569482495	1.44941328958356
C	2.67002419827755	-0.72090138892698	1.38379954310665
C	4.60045821158595	0.79982396621785	1.21071701417577
C	-0.11359173894963	-1.96057244980758	2.70153347409342
C	6.67651826072803	1.27251140704111	2.28172353871618
H	-1.44104178037844	-3.29276177262010	3.71412780299354
N	1.33486328255413	-0.52576108520435	1.37384525943397
N	3.19924188091996	0.51631487419539	1.28456915443792
C	5.31684894909667	1.00494287652563	2.39004999414447
C	0.36594727050730	-1.30199657216371	3.95739277860534
C	1.03239411910125	0.81606734196705	1.27346568274450
C	2.20836018104341	1.47340573176685	1.21611531218435
H	7.25090331300367	1.42726703676623	3.19014872909337
H	0.01633528979491	1.17270925700830	1.25193930559637
H	2.43195706006061	2.52360689281235	1.13170693395095
C	4.65356168523019	0.93509736734257	3.73015228550155

H	1.45584772951155	-1.31392790475430	4.02523242780539
H	-0.03942896733408	-1.80898260090511	4.83292073988927
H	0.05264136270380	-0.25458825568366	3.99599632642716
H	-3.23112801720032	-4.67248857247413	0.81903920932731
H	-3.13300410630456	-4.63504759208681	2.58981949434649
H	-2.04465105374382	-5.66435803055816	1.66084814818597
H	-0.06126021673361	-2.26924037970697	-1.86817370816075
H	1.45034864351498	-1.69590236425552	-1.14783479823946
H	0.06579126376661	-0.61768969667866	-1.25009751736658
H	3.81953303010800	1.63825552832920	3.80077765039457
H	5.36668463501245	1.16882154394227	4.52056927250228
H	4.24890749762741	-0.06293348834531	3.91835486775550
H	9.19573664835553	1.34210263226270	0.00435672994386
H	9.33513066105462	1.16071557522683	1.76281181622799
H	8.95340303012180	2.73071118070165	1.06509580559838
H	5.03685915507035	0.81145617378702	-2.17707434129069
H	3.57205197846159	1.37906585829651	-1.36611347217202
H	3.97639353360994	-0.33143772014623	-1.34035240871432
C	5.06925756448339	-4.82471180556136	6.29389593512727
C	4.30286293595441	-3.66826520872084	6.53818039953637
N	5.29692830127031	-5.11199650317850	4.96755115825885
S	3.87942687939797	-2.95169898375361	5.01961162854384
C	4.74399079623122	-4.23052949806448	4.19100937926777
S	4.85572166742605	-4.30399166032716	2.46685591526698
C	5.53077035521545	-5.57758890293715	7.37433365560271
C	5.22213661166198	-5.16870247391345	8.65931857588937
C	4.45956428396734	-4.02032394529907	8.88682629550698
C	3.99195824721391	-3.25821621111608	7.82798260169847
H	4.22982846671075	-3.71981801434256	9.90267146972596
H	3.40043587567268	-2.36716868003068	8.00355362703401
H	6.12169056060004	-6.46866487384811	7.19456838513629
H	5.57679591172938	-5.74851776229170	9.50402135253973

H	5.01002861267594	-4.19444619933359	-4.06278565276731
H	3.13169765953926	-4.68115835080644	-2.59626081509701
C	5.18389258222808	-4.15391857130813	-2.98046395653914
H	5.14602040442147	-6.14571678537901	-2.60587409337348
H	3.38964724246872	-2.93957002541522	-2.76719997201533
C	3.81019716823879	-3.86010487847277	-2.35930974313558
N	5.81493886108776	-5.38524022729233	-2.56829882860296
O	5.75377598395309	-1.90836610201645	-3.40893981171484
C	6.13091892616467	-2.99947776272528	-2.74280485377065
H	6.36524018395130	-1.18763479458891	-3.19048905898365
H	6.14239129597748	-5.31196844873663	-1.61152957848402
O	7.10340673786692	-3.03855606754684	-2.03290025611021
S	3.80262699143275	-3.62065892757245	-0.53966657243679
H	4.24711113653880	-4.85020759844185	-0.23041999727092

AuIMesStzn – TS1$^{SPT/NPT}$

Coordinates from ORCA-job

H	7.40754523139328	0.64747066306383	-0.57586039644588
C	6.76152622880241	0.76034140355994	0.28945269571641
C	4.85129373627617	0.00551655119092	-1.15431549752222
C	5.40926690270653	0.46558922734500	0.15611472315505
Au	3.55729790855208	-2.64126514619324	1.73861030241393
H	-1.57582146204609	-3.62389938212048	-0.76396559106813
C	-1.18401092766470	-3.18383215365068	0.14802855096770
C	8.76893392242060	1.48118678246410	1.61720176973261
C	7.30232089895563	1.20021041513500	1.49294355816727
C	-1.68455447552572	-3.59872192894209	1.37775771870004
C	-2.73178195710862	-4.66734989147892	1.45962188517376
C	-0.19320904090830	-2.21361167986200	0.04875476634956
C	0.30993015556125	-1.76838308131061	-1.28954556388836
C	-1.17872906356995	-3.00956986519455	2.53123303402651
C	0.29788930648926	-1.66264529996517	1.23227777625256
C	2.63031919519396	-0.86664811390939	1.32223135933382
C	4.59690970349686	0.62301354441518	1.27847267710545
C	-0.18736544517634	-2.03559150618744	2.48554138924795
C	6.45273224182037	1.35691122464357	2.58292830656529
H	-1.57050198219187	-3.30859416233991	3.49867070049059
N	1.30850535697495	-0.65249487348789	1.15329029877391
N	3.19697691321642	0.34807304407543	1.16509015626593
C	5.09284160700138	1.07909522773410	2.49967742849219
C	0.32804150457875	-1.41102892599618	3.74454242284087
C	1.05158142789426	0.67745371738430	0.89460856901250
C	2.24290577507650	1.30870364577636	0.90251285839486
H	6.85533900958820	1.71042072593751	3.52723315862781
H	0.05285423072769	1.04631970910314	0.73180025767139
H	2.49986102948033	2.34321519778241	0.74838060367796
C	4.19680783186558	1.28380607171657	3.68182866405376

H	1.37354124564326	-1.68109565891537	3.91900076291977
H	-0.25755425009070	-1.74429169588826	4.60140831071467
H	0.28155994777548	-0.32004148199534	3.69784252796269
H	-3.40721133174866	-4.62833514646630	0.60298254280595
H	-3.32016337399612	-4.57422881643647	2.37385222521280
H	-2.27004474742566	-5.65977907263701	1.46553237268647
H	-0.06106929206547	-2.42772712790360	-2.07421294986781
H	1.40174657765389	-1.76345901262349	-1.32878712526080
H	-0.02297103070096	-0.75161307958162	-1.51760040179657
H	3.52242946542810	2.13050227564259	3.52320834379538
H	4.78664693713536	1.48590755602128	4.57587048233800
H	3.57413911691922	0.40652688065152	3.87010660411072
H	9.19511116487008	1.79120914500649	0.66154102625631
H	9.30690929272692	0.58419944841320	1.94012560687517
H	8.96171028939076	2.26087533839392	2.35617715852492
H	5.62740501767761	0.00746675928354	-1.91994760382413
H	4.03590071124004	0.65144192192614	-1.49050924040956
H	4.44838349979995	-1.00807156245958	-1.07981314837415
C	5.43699603361499	-4.23348521183990	6.67465959095428
C	4.49252975293682	-3.03720469884206	6.81954396135276
N	5.28546876902694	-4.83128651159634	5.35944060216651
H	5.24165079137125	-4.97617926896913	7.45193228098310
H	6.48065253076437	-3.91276058913183	6.78717824408371
S	4.27171474431703	-2.43729305200788	5.12498935917365
H	4.90989978152960	-2.24877159475013	7.44482054581437
H	3.51968620762427	-3.33194222859054	7.21660592707811
C	4.78667195028734	-4.02390605629924	4.51265252676398
S	4.64480664312389	-4.40778531986004	2.82047983091747
O	6.52022081810527	-3.65236218731141	-2.49396470956046
S	3.57128283610702	-3.98142977620774	-0.24929549130661
H	4.04870773975454	-5.15400211030571	0.19892490731101
H	5.71611416678458	-5.81794897317525	-1.40325264890990

H	5.42348017195663	-2.02248755824045	-3.69507201313519
C	5.40372535589568	-3.74388786542916	-2.93679420052191
O	4.81302670303332	-2.77385145309761	-3.63595362676855
N	5.25534113204203	-6.06287236013602	-2.27286892502440
C	4.49456396553886	-4.94120571913289	-2.77100588995398
C	3.24617466977867	-4.56171236767979	-1.96277081182647
H	4.63246163570647	-6.83819626013731	-2.07675410539744
H	2.71021957330385	-3.74201641907829	-2.44267964443758
H	2.57890678062265	-5.42324845984053	-1.91082105466773
H	4.12935224265497	-5.18814176945843	-3.77601073204578

AuIMesCl – TS1$^{SPT/NPT}$

Coordinates from ORCA-job

H	6.74150920530632	2.27840317347081	-1.20613945372499
C	6.09505224962066	2.44369102063233	-0.34965043014077
Cl	5.43176179012359	-2.71898153949596	2.10673613579396
C	4.38677454653960	0.98947159071565	-1.47710611740953
C	4.83725518063472	1.85002809176779	-0.33827219439339
Au	3.76382889007402	-1.05846999150820	2.04142168253952
H	-0.52480701078631	-4.07987683131627	0.31352734338494
C	-0.43777352968886	-3.24317706439303	0.99992287592232
C	7.91201545230022	3.84488298077672	0.67497696197197
C	6.54047889365894	3.24117041339549	0.69843586387313
C	-1.04930049448835	-3.31659419776336	2.24637727641089
C	-1.79213312730671	-4.54787103723119	2.66710380564486
C	0.28422378070303	-2.12333034918998	0.59958682155306
C	0.92757920250238	-2.07021612086612	-0.75151150025003
C	-0.93898788773833	-2.22629448374244	3.10391076073312
C	0.38219360153769	-1.06123183405763	1.49716614751104
C	2.44573061271286	0.26126505225293	1.28640188631517
C	4.02224705706956	2.07901675222940	0.76945229912537
C	-0.22853478487704	-1.08517036507881	2.75122932468674
C	5.69055766702770	3.44941486362436	1.78039533723611
H	-1.42016913707581	-2.26147893686283	4.07692416624390
N	1.11758898126202	0.10573189850550	1.11529821689173
N	2.71804752053516	1.48981313661472	0.80162798570188
C	4.42433616795442	2.87915568038763	1.83910576317812
C	-0.12760236242696	0.07833322660458	3.68792326567235
C	0.56330900564313	1.22470992048833	0.52801252894326
C	1.57250700926778	2.09708890888627	0.33013095393124
H	6.01896859318810	4.07570893432667	2.60429601054473
H	-0.48881720397867	1.29313598824622	0.30837937569082
H	1.58376940986695	3.08495693531492	-0.09877112773804

C	3.52912099678079	3.11605152673794	3.01595378062863
H	0.91506080080694	0.31969786440353	3.91137937353335
H	-0.63638324145699	-0.14277146482304	4.62568249475790
H	-0.57944077273094	0.97651412904414	3.25818675867651
H	-2.10600880305856	-5.13612373613667	1.80354146015347
H	-2.67476589040069	-4.29554313991306	3.25822782479674
H	-1.15693771553767	-5.18633004109750	3.28920633498426
H	0.72530698296716	-2.98652884530569	-1.30597710922902
H	2.01130393727558	-1.95107638659520	-0.66848251259820
H	0.55571939918720	-1.22559928451610	-1.33779621433110
H	2.59598560271903	3.60140161359296	2.71795072287318
H	4.02486495032822	3.75154409301089	3.74956262758215
H	3.25956096681241	2.17471742759865	3.50280671665546
H	8.28757647338875	3.93525281547941	-0.34544248379616
H	8.61733060835589	3.22003203477541	1.23200891953265
H	7.91797394779479	4.83293016958982	1.13901179455537
H	5.13080749368548	0.99125750920852	-2.27357621905044
H	3.43735733847373	1.33666558597139	-1.89267479009514
H	4.23565423258343	-0.04366127149011	-1.15082074352415
H	5.12667823305837	-2.40844721920651	7.50996779791081
H	4.42201201466071	-3.40628684705942	6.42316564610733
N	4.26384152423238	-2.88765668290731	7.27991889038740
H	4.91986972762754	-1.88647188984708	4.65491389002609
H	4.41907001722068	-0.20963670292199	6.63739483696659
C	3.20227606962010	-1.92571706903169	7.09743347996049
S	3.85609635061647	-1.09158426621675	4.45843452666246
C	3.54014583893111	-0.70761016722342	6.22514863059488
H	2.92710415734993	-1.51910179043297	8.07887758173706
O	1.95085023774749	-3.75065011401489	6.12988808100079
C	1.97237498713035	-2.62862848081483	6.56797787284208
H	2.71349581130371	0.00403722868114	6.21991669509509
O	0.89466997499724	-1.84710106852329	6.62921943974307

H 0.14339846836426 -2.32778134675045 6.24743192901365

AuIMes2tu – TS2^{SPT}

Coordinates from ORCA-job

H	6.59366054321471	2.44827997665963	-1.86929189267459
C	6.19515801744104	2.28933205852114	-0.87210587958879
C	4.09204304650895	1.44235172017531	-1.95174380178127
C	4.90868107352412	1.77710164632282	-0.74203125320189
Au	4.01702105620786	-1.79767315344984	0.78701862025671
H	-0.94669280838035	-3.79211289931241	-0.64618772994273
C	-0.56233836659571	-3.26560024907641	0.22199431394591
C	8.37258595909734	3.12139470593757	0.06573764396057
C	6.97785904729256	2.60082258635695	0.23410894991953
C	-0.82449198530339	-3.76249646807141	1.49411686376331
C	-1.59508959367165	-5.03417369083781	1.67672538373372
C	0.18125654052611	-2.10740008675723	0.02473948669519
C	0.45430638160573	-1.59673770866769	-1.35608558214857
C	-0.33483849785086	-3.06443164145688	2.59339926688651
C	0.66284128462696	-1.44827876047588	1.15502802827519
C	2.77091907688991	-0.22494550315393	0.83433210432996
C	4.41682163520347	1.58480950029196	0.54808559476496
C	0.41196205173298	-1.90114225779811	2.45024759870660
C	6.44354988716892	2.39773300412079	1.50251475289705
H	-0.53944995110414	-3.43235089584241	3.59413287890496
N	1.43277557837532	-0.25300891708299	0.98225648270710
N	3.08681118501813	1.07842140490010	0.71326677205590
C	5.16260864538893	1.89076847907372	1.68661411217615
C	0.92506621128121	-1.16581139548042	3.64965863397478
C	0.91227714584995	1.02224999302032	0.95353688186598
C	1.95549315977637	1.86186941933332	0.78330190600421
H	7.03704515753364	2.64379464306732	2.37771151697385
H	-0.14299758365863	1.21156485732615	1.05599851097815
H	1.99867358542063	2.93526744247360	0.70602146545986
C	4.61098386846173	1.68164969795507	3.06282354309745

H	2.01614835886386	-1.09640693255662	3.63694824306685
H	0.62579197685325	-1.67608852058757	4.56491066162472
H	0.54033295918752	-0.14322524502957	3.68639607132560
H	-2.26822817531941	-5.21426613036162	0.83703814222375
H	-2.18042916195269	-5.01440841989945	2.59775207754761
H	-0.91429084610217	-5.88898764083317	1.74114079065314
H	-0.03639761849148	-2.22603342341775	-2.09831667050386
H	1.52694124937547	-1.58902457907416	-1.56934232498990
H	0.09518362946305	-0.57217348745248	-1.48369963126008
H	3.68622579317736	2.24467063638909	3.21416116692942
H	5.33260717429830	2.00121095166842	3.81440016625582
H	4.37758525002442	0.62803149627690	3.23857881906118
H	9.09652846878036	2.30174658877099	0.11327212716387
H	8.62950855005262	3.82867805680457	0.85649054482995
H	8.49785946309628	3.61410535619805	-0.89973037004940
H	4.63982116069511	1.69158272494488	-2.86039807801992
H	3.14499358447022	1.98791729641409	-1.96221663030755
H	3.84863911523676	0.37647715147399	-1.98090601176877
S	5.43349119881766	-3.60308800376986	0.56074424673782
C	5.59856023398793	-4.62928222989969	2.06011129685339
H	4.78379588334388	-5.35184607224827	2.01899422658107
N	4.25682021581500	-3.45979634331057	3.76404296880261
O	6.51608720869761	-1.75312808770779	3.76526518657568
C	5.56730610404553	-3.95670763791987	3.43049033473172
H	6.53900347221516	-5.16377249256172	1.92203041781748
C	6.68269485115530	-2.93166924497711	3.58081129597066
O	7.88664282869963	-3.49895119648732	3.50468549913266
H	5.81263146050776	-4.76088378458604	4.13501919040283
H	8.56354403412367	-2.80936279050889	3.59498718887460
H	6.86283219059512	-3.02095703725430	0.48585399479914
H	4.02735280630124	-2.65787056001283	3.18135037401117
H	4.25114380774971	-3.12134142720417	4.71969524231181

S	8.53349983743336	-2.41904997348384	0.33162340110377
C	8.37806126206020	-1.66610947676079	-1.21310244056867
C	7.23075991058538	-1.68313593094899	-3.37214353997322
C	8.92020932421109	-0.08952979976593	-2.75960758403556
C	8.03373688077455	-0.54563906055651	-3.68733715279300
N	9.10475394714571	-0.61434010572269	-1.52587004899533
N	7.49087933729813	-2.18908137695098	-2.10282465272829
O	6.38743954289433	-2.22534447610421	-4.08392381910359
H	7.92571946204174	-0.07649479443845	-4.65567853015412
H	6.97874623798620	-3.02361207939548	-1.84864336192755
H	9.54345567821946	0.77044059477632	-2.99060800120598

AuIMesSpym – TS2^{SPT}

Coordinates from ORCA-job

H	5.48525322350151	3.35475807946254	-2.17161107524138
C	5.37089830571749	3.03447529528892	-1.14050901962415
C	3.05912523751842	2.25284895569645	-1.73602717731703
C	4.17105596035559	2.44820490044709	-0.75254946032710
Au	3.83481491502323	-1.31799566165014	0.39757420015399
H	-1.26728343989467	-3.45087134287528	-0.05480033378977
C	-0.72509023067362	-3.00526762900577	0.77354030756157
C	7.71345233852433	3.82734262536485	-0.69715952908342
C	6.41926799998392	3.22194379804955	-0.24662874684005
C	-0.72639207378752	-3.63969706104565	2.01054868424384
C	-1.43525467746550	-4.94512480485106	2.20468799458878
C	-0.05221886614539	-1.80596692924717	0.56300339486117
C	-0.06912952232459	-1.14392953920324	-0.78003084380814
C	-0.03641198362481	-3.04457149884623	3.06230087552879
C	0.63275437808697	-1.25126313620062	1.64285732698442
C	2.57151300335508	0.12396053123326	0.99379266678595
C	4.04642617158621	2.04752722522351	0.57686026625214
C	0.65436690954612	-1.84979201099569	2.90307617402944
C	6.24703334080952	2.81511699718063	1.07271992636956
H	-0.03159845598749	-3.52307896733203	4.03687671946821
N	1.31660367254796	-0.00598534683767	1.46435061816907
N	2.81719553046555	1.44845527755650	1.00261687541867
C	5.06767542068047	2.22428112472300	1.51067030399859
C	1.39859408314413	-1.23467579920075	4.04773272132314
C	0.77958257845949	1.22732480215701	1.76645613464402
C	1.72533283027701	2.14434712267119	1.47437319624030
H	7.05217751509827	2.96284683677248	1.78598616898158
H	-0.21794615089806	1.33508560786447	2.15801174833864
H	1.72346535432885	3.21818841290612	1.55732210100060
C	4.90943982448206	1.79103689579356	2.93518424782934

H	2.45668720701618	-1.10169512697922	3.80574965794182
H	1.32613097045857	-1.86741831235757	4.93210357888745
H	1.00136894013331	-0.24800206667140	4.30036628240000
H	-2.20177813155438	-5.09778071877294	1.44349422251517
H	-1.90429009962318	-4.99819511359700	3.18917973830961
H	-0.72963727295937	-5.77915158460484	2.13673102871006
H	-0.73465687740289	-1.67782254515528	-1.45814801226683
H	0.93011906983891	-1.12841483193339	-1.22473911651323
H	-0.40580465651354	-0.10633475387873	-0.71276186911988
H	4.04279098294835	2.26374820420641	3.40455719993926
H	5.79620487167721	2.05139835275974	3.51271112514164
H	4.76162440603095	0.70950918797238	3.00490666704504
H	8.43208676531482	3.04499800240158	-0.96093494860425
H	8.16517503881065	4.42973851813535	0.09316444598017
H	7.57173495930528	4.45455467558249	-1.57864977026855
H	3.34100992042200	2.64755172708078	-2.71193706257758
H	2.14478290624834	2.75759569316606	-1.41350315040588
H	2.81569725821638	1.19294560058091	-1.85217372599927
S	5.33524095244304	-2.87228051538091	-0.40277881149949
C	5.19217185498193	-4.49269484381554	0.41959922737676
H	4.35814302515841	-5.00413159043975	-0.06111666004177
N	3.66461110008860	-4.10594645340366	2.32100785405648
O	6.01362094757365	-2.85957065557786	3.31984868225497
C	4.97762963136720	-4.55310879533273	1.93161068680636
H	6.11423006506849	-5.00958730602753	0.15221903964963
C	6.11689089055028	-3.87226479419648	2.67489811173408
O	7.25983748736766	-4.54294341866115	2.53878533310598
H	5.05670981281966	-5.61875714457102	2.17693553152370
H	7.96708708294994	-4.04357413032063	2.97647234654942
H	6.72761421961511	-2.37898182228208	0.07003431137154
H	3.58491425966952	-3.09891321681677	2.20154071577232
H	3.52010653593175	-4.28259314654380	3.30879937606282

C	10.75206484755046	-4.63584685829783	-1.87918638697370
H	11.34079745058477	-5.34611745196472	-2.44373185429012
C	11.30779692141363	-3.50098585173594	-1.31341456072016
H	12.36882118622425	-3.28821430110747	-1.42043214132759
N	10.59857770045849	-2.61892635465005	-0.62126532610319
C	9.28534775190327	-2.86684784281365	-0.47500206423447
N	8.65803841788733	-3.94225166293650	-0.98031209849871
S	8.35836930109770	-1.71328299135008	0.43702810227814
C	9.39369834812854	-4.80241499196280	-1.67304124306691
H	8.87095075810258	-5.66563352884476	-2.07824092964278

AuIMesSbtz – TS2^{SPT}

Coordinates from ORCA-job

H	5.76859666734943	2.59542087957724	-3.21126045939782
C	5.66741674190214	2.51810161450032	-2.13306567978111
C	3.40735464753477	1.48927355908542	-2.51221075056394
C	4.50114411984665	1.96981940495650	-1.60935619911443
Au	4.27592389610318	-1.46737891198818	0.40977871816468
H	-0.95118501807979	-3.62884891176409	0.51061547095274
C	-0.35603818145125	-3.03606932639780	1.19844926785531
C	7.92649804250877	3.59555600456951	-1.90067975596758
C	6.69779520374767	2.96887936597986	-1.31577369357374
C	-0.28326696511321	-3.40956071553762	2.53570910637136
C	-0.98453560096593	-4.63764020333260	3.03039690035598
C	0.30731595095808	-1.91457021348956	0.71184292951594
C	0.20266165279788	-1.52754901075065	-0.73098059366769
C	0.47478507513186	-2.62816898381893	3.40197262223989
C	1.06410828531273	-1.16715237915610	1.61305244934097
C	2.98220029918796	0.05282712344658	0.65003995281614
C	4.39396125346269	1.87791056965627	-0.22260732366244
C	1.16066242702573	-1.50176141301727	2.96407848688619
C	6.55157431782656	2.84947313343875	0.06290992978089
H	0.53665766129562	-2.90072425241398	4.45119042659816
N	1.72829855998824	0.01155228490624	1.14592169344121
N	3.18957233768453	1.34974424233142	0.34208073874040
C	5.40653507258214	2.30893139163413	0.63518941870295
C	1.97821081244313	-0.68386082827618	3.91526751017517
C	1.15801673654483	1.26737929196033	1.14783491751599
C	2.07975417975204	2.11138587457071	0.64197600772780
H	7.35034689958752	3.18865260515054	0.71565107766387
H	0.15714604441749	1.43998061987438	1.50634415212361
H	2.05137237791732	3.17395957462233	0.46823818677987
C	5.27564982227657	2.18848637884504	2.12187490412796

H	3.03214601008028	-0.67678349794487	3.62298153172070
H	1.90632116799624	-1.08953025977836	4.92425532440831
H	1.64561501102189	0.35711060054696	3.93949163358954
H	-1.37061601474682	-4.49294113477613	4.04129519992576
H	-0.29412308753844	-5.48639738450675	3.06493211259083
H	-1.81275048428698	-4.91315483317831	2.37570535741118
H	-0.43121793892661	-2.23229428262754	-1.26893360283066
H	1.18544081391983	-1.51401070894854	-1.21017122056387
H	-0.22335952357280	-0.52734554455497	-0.84660716721810
H	4.37227376469035	2.68331031071320	2.48746029908076
H	6.13668703187316	2.63699947658220	2.61717063677889
H	5.21286287364098	1.14021576239375	2.42765213063453
H	8.82137414000327	3.30324268951883	-1.34760872981688
H	7.85976326997627	4.68715746001245	-1.85577275796814
H	8.05543451732482	3.31509958698646	-2.94705113118969
H	3.66511029978387	1.67453325965216	-3.55483316428347
H	2.46032211780228	1.99173746524034	-2.29912712714998
H	3.23510951179036	0.41649306763082	-2.38664450995883
S	5.85167170353977	-3.11011665383545	0.07511204095067
C	5.07436427786507	-4.74275473189220	0.33534165231197
H	4.11120362246074	-4.77427588114635	-0.17342685344388
N	3.82248691319506	-4.37550338749781	2.41757690176957
O	6.40819471909756	-4.48218670120477	3.53480074029741
C	4.86233741407890	-5.15197695105126	1.79128307860373
H	5.73720427703529	-5.46164660930589	-0.14628391741553
C	6.18029208527512	-5.14252935029707	2.55171229626656
O	7.06815297602545	-5.98620902803140	2.02426337751921
H	4.54373182966140	-6.20244656660034	1.76989351354490
H	7.88178916647546	-5.94430452587320	2.55075814725836
H	7.42609502597894	-2.38752685892100	1.70751058343077
H	4.07914660690907	-3.39223968382714	2.40699018340199
H	3.73022458115367	-4.63562470055472	3.39282345450320

S	8.27301600577145	-1.79393537548376	2.60087508786720
C	9.20444235254451	-0.89687339782039	1.43966414721918
S	9.01871362557524	-1.11999689677193	-0.28363370290261
N	10.09821628593364	-0.03939911538473	1.80655372955493
C	10.26308097172285	0.05596147676858	-0.53703437698723
C	10.71908384041024	0.51962011547405	0.71047218887505
C	10.79226644294653	0.52994259428028	-1.73056186399145
H	10.43357325407798	0.16628360821075	-2.68609756302429
C	11.72795466446662	1.48117822155899	0.75720277074588
C	11.79432854360550	1.48307351249854	-1.66246701445448
H	12.08213007304444	1.84235725729787	1.71583258053141
C	12.25596397791673	1.95367387797149	-0.43002123492371
H	12.22510457680464	1.86814113253619	-2.57947824953733
H	13.04081338604746	2.70100381690989	-0.40571692520244

AuIMesStzn – TS2^{SPT}

Coordinates from ORCA-job

H	7.02922462644684	2.41345315245190	-1.45634506597392
C	6.57404318185004	2.22619225593564	-0.48854172372440
C	4.48010189459943	1.56019987273056	-1.70530833313473
C	5.25852343245180	1.77966978411690	-0.44509552558250
Au	4.17766393387405	-1.81011080878255	0.89878172318281
H	-0.80649353121132	-3.50456433467326	-1.00385866597908
C	-0.45963358596480	-3.04493936932208	-0.08357200714898
C	8.74601725918590	2.87915678233515	0.59051481142588
C	7.31686799637704	2.43670499776170	0.66880755138461
C	-0.83763068755607	-3.59086108442523	1.13712458433825
C	-1.72988156135822	-4.79293470823339	1.20171711163545
C	0.35594252051598	-1.92024950962264	-0.16487944987442
C	0.74943651251044	-1.35389868172913	-1.49382542645883
C	-0.37797715745610	-2.98782837894683	2.30469844024206
C	0.79347278508915	-1.35100167036632	1.02900467769226
C	2.97037203139209	-0.20647810277636	0.91823118695504
C	4.69489496818947	1.54484221489462	0.80892526808439
C	0.43736943104682	-1.86379321163351	2.27733081018079
C	6.70931146858749	2.20342089402962	1.89782227239785
H	-0.66039296137337	-3.40336404013326	3.26732504426376
N	1.62438094772412	-0.18605334696915	0.97436522423662
N	3.33942885116309	1.08900002755465	0.87975902507476
C	5.39593702731179	1.75720553033063	1.99525220268924
C	0.91843969632108	-1.23247449282506	3.54690207033311
C	1.15385907945846	1.10954802636826	0.96956616603767
C	2.23587107919576	1.91377451027093	0.90968725759018
H	7.27090403977237	2.37514208848837	2.81102470143565
H	0.10166437642242	1.33609629351842	1.00952873593967
H	2.32354090528495	2.98694886253863	0.88565693653421
C	4.76350479915295	1.51711188724289	3.33116408968627

H	2.01059678972036	-1.22246252482756	3.59533250366284
H	0.54264331551941	-1.78105513639166	4.41042167904863
H	0.58369036182677	-0.19494591774252	3.62960103051971
H	-1.85430510824744	-5.24719657958186	0.21797407394705
H	-2.72161955435590	-4.52015590150243	1.57456551790916
H	-1.32704733385189	-5.54595712978873	1.88319109533873
H	0.30275102124295	-1.93353706658904	-2.30146006653692
H	1.83519871381373	-1.36478194297780	-1.62297328655141
H	0.42475447508726	-0.31517963268955	-1.59846665371622
H	3.86812586257544	2.12986639763842	3.46505636590945
H	5.46356212845067	1.75536623664605	4.13174255783023
H	4.45757867149904	0.47325414354723	3.44331598954878
H	9.04529837131317	3.41656791789598	1.49175542816026
H	8.91463949974263	3.52310498218724	-0.27455990086769
H	9.40915175122303	2.01427760863810	0.48687844731608
H	5.10104452212620	1.76809769043052	-2.57654736945129
H	3.60103496778138	2.20878394150693	-1.75132808644821
H	4.12211903756526	0.52953369531170	-1.77657244554281
S	5.58575612218165	-3.62730424495365	0.67256980034156
C	5.68358829552388	-4.58431682211853	2.21697777651895
H	4.81399195318379	-5.24196435383125	2.21406071277351
N	4.47695033539926	-3.08587761855997	3.79119915948745
O	6.87836550210675	-1.75098530736157	3.88796945047878
C	5.69504964091282	-3.82499547087400	3.54879190920931
H	6.57622645483891	-5.20440983384262	2.12266033490255
C	6.91613182059634	-2.93834250133514	3.68135280013666
O	8.04723662279261	-3.62671847392390	3.55859584601487
H	5.82719749852179	-4.60783873701584	4.30690295265403
H	8.79558413406908	-3.01378132652183	3.63440833580703
H	7.00493034912882	-3.03798322181512	0.59466029254873
H	4.69592595367434	-2.11246159701877	3.96900836970457
H	3.99851700028015	-3.44376628093794	4.60699841808530

C	8.97176721916010	-0.40106533191699	-2.96120760618818
H	9.73149799268325	-0.90068558868143	-3.57652643298217
H	9.06737112143836	0.67311734686132	-3.13792411622242
C	7.57341286683877	-0.88654442214391	-3.34551913834835
H	7.50939647499236	-1.19454850983044	-4.38852592587502
H	6.81090137011405	-0.13232075551480	-3.14445910107038
S	7.29823605538365	-2.30488367074324	-2.26044544349862
C	8.52088693897530	-1.66012880866581	-1.12201101039727
S	8.66712610404329	-2.39853173419512	0.43344957102412
N	9.23864132107371	-0.70399895703325	-1.56551752880217

AuIMesCl – TS2$^{SPT/NPT}$

Coordinates from ORCA-job

H	6.49370989026855	2.57235086113676	-1.60286407994047
C	6.09450211966862	2.39162440460125	-0.60955486977170
C	3.96753885871998	1.63113037659462	-1.70654340783848
C	4.79597130864437	1.90845225542134	-0.49053666077009
Au	3.93616059618438	-1.66160169985507	0.84105033827693
H	-1.16851062670989	-3.58507462090958	-0.54185630819523
C	-0.74651556780233	-3.10644250543379	0.33683005438196
C	8.29874110480535	3.12714069569229	0.34505653237841
C	6.88859731469804	2.64642220904519	0.50327759842506
C	-0.97507347260376	-3.65894733536153	1.58772119335662
C	-1.77340144164096	-4.91704688086629	1.74391730031995
C	0.01197931472999	-1.94874682789146	0.16619745228044
C	0.22121214547225	-1.40096084692717	-1.21303075699424
C	-0.43114380260688	-3.02349274676168	2.70331123420324
C	0.54233487826173	-1.35293368834471	1.30611944329535
C	2.65479465362750	-0.12401313837151	1.00511143382314
C	4.30458413206475	1.68502823242344	0.79493482060203
C	0.32815136158920	-1.86948463204269	2.58924265055667
C	6.35214568268437	2.42079924532852	1.76664612247770
H	-0.60492962758251	-3.43814056017396	3.69170515933414
N	1.32004061807136	-0.15706717879742	1.18000147565060
N	2.97149453741719	1.18436900019756	0.95094084726274
C	5.05969102147413	1.93957376494484	1.93963594009707
C	0.89491008928464	-1.20178961240806	3.80391047792530
C	0.80371560098320	1.11921987064540	1.23452494118402
C	1.84549036688110	1.96544756443925	1.08927931585460
H	6.95468719737138	2.62446124415277	2.64649616664111
H	-0.24874335698820	1.30407052546994	1.36875599993884
H	1.88981949482757	3.04145440754139	1.07094554335670
C	4.50713179212588	1.69945972246894	3.31037924516154

H	1.98218782820002	-1.10981015930154	3.73842999065058
H	0.65321435953556	-1.77467117169499	4.69899926704448
H	0.49528384120102	-0.19150368145606	3.92818675104889
H	-2.28374339866658	-5.17984793606889	0.81657223669121
H	-2.52038290775621	-4.81496675993732	2.53471280699196
H	-1.12525832568817	-5.75406719949564	2.02037530088649
H	-0.74037608571705	-1.17178613541978	-1.67942719329349
H	0.71640014284584	-2.14257064350797	-1.84477898898057
H	0.82459765603112	-0.49493089976553	-1.22013223237914
H	3.60338539923354	2.28854952212519	3.48727176185853
H	5.24254774347259	1.96615953995114	4.06915503115123
H	4.23728627425166	0.64896237912709	3.44932664443824
H	8.60219222882182	3.75127340119158	1.18730420659203
H	8.42254696053212	3.69737915639347	-0.57711766015248
H	8.98904114717412	2.27862608172247	0.30100821224801
H	4.50189356356718	1.93313822527283	-2.60715084354588
H	3.01534985308730	2.16668862077472	-1.67658193745194
H	3.73375743954524	0.56582550627584	-1.78993172853285
S	5.47354507415868	-3.34986663476795	0.49755803142950
C	5.53733804248413	-4.55844893892428	1.86258532942935
H	4.72090050494243	-5.25876425435603	1.68529000176054
N	4.10332702395239	-3.60751039655878	3.62837976498107
O	6.35029994314921	-1.89767082788004	3.88739230266241
C	5.43165939955429	-4.06128840523038	3.30295658144814
H	6.47969316207133	-5.08685322654966	1.71743032467894
C	6.53159073058325	-3.05807889377912	3.61809291433856
O	7.73768539642802	-3.62175858966676	3.57275856355067
H	5.64682742726287	-4.94342868780304	3.91776620078643
H	8.40753995916696	-2.93660431304004	3.72430065120031
H	6.85944701856859	-2.58939338305225	0.61191402742978
H	3.89759573804371	-2.74277999140921	3.13284932094937
H	4.04999275963335	-3.38236929471715	4.61560291977760

Cl 8.29202791640558 -1.78577411440999 0.54865623703663

AuIMes2tu – TS2^{NPT}

Coordinates from ORCA-job

H	5.29352566855522	0.97875581689237	-2.45542773370271
H	4.41213664394927	-0.09796148771473	-1.36320827681738
C	4.64799237121420	0.94834779811824	-1.57784294527505
H	7.15925280125450	1.92823724647691	-1.45069557407366
H	2.13334456270617	-1.90663391209443	-0.81633401851771
H	0.63799306674996	-2.61739385501963	-1.43934351507995
C	1.04182246987851	-1.84395353929236	-0.78632074082804
H	3.70626765266046	1.44442802325314	-1.82573470355073
Au	4.25005269650556	-1.43554964948396	1.73942467103221
H	-0.51305596483974	-3.83046651452273	0.17141888322408
C	6.63548951392898	2.04889762868213	-0.50742390548132
C	5.32324553838055	1.59829248805840	-0.41007630563598
C	-0.26743735902198	-3.10800406516893	0.94381470578641
C	0.52826297811510	-2.01845270931341	0.60933407372360
H	8.99488839820526	3.25001094273731	-0.59626998354538
H	-2.20757580254147	-4.79769623083886	1.72513439887292
H	0.76577646240379	-0.86894680507810	-1.19597832382538
H	-0.95036315847114	-5.34006915594388	2.83251743437320
C	-1.59007419038419	-4.49107510838001	2.57137409101597
C	2.97808711833667	0.05352600373154	1.30156882034103
C	-0.75935425598021	-3.29106491496652	2.23228199694834
C	8.71539946668618	3.08846493059346	0.44588220736604
C	7.28862095502966	2.64644470494558	0.56476081011978
C	0.82641346683321	-1.10112164043967	1.61721914654912
C	4.67070176219202	1.76582082415563	0.81046591708246
H	9.38879396464306	2.32822964679812	0.85487947071821
N	1.63192046250051	0.03862811605829	1.29624024366081
N	3.32046849345286	1.30482010671390	0.93934749477369
C	-0.44496530857061	-2.34594445725433	3.20245263941483

C	0.34798822627142	-1.23900939955087	2.91961708963540
C	6.59378776810349	2.80488348474975	1.75992248286863
C	5.28152926607236	2.37196645405473	1.90873576632429
H	-2.23902554696435	-4.29410783482911	3.42608924010060
C	2.19792615420584	2.06990263377180	0.70834980356083
H	8.89242952939381	4.01053656570583	1.00298850873033
C	1.13321628423877	1.27165631903926	0.93344693496823
H	-0.82719640657294	-2.46885696300758	4.21110465392521
H	1.75172301700421	-0.11058396506370	4.09603813730370
C	0.67173608416588	-0.23608283984310	3.98323317104876
H	7.08565228838491	3.27703352936495	2.60482821174456
H	0.07543075409888	1.46340995864018	0.86841918115157
H	2.26083159772534	3.10175898406963	0.40626539620189
C	4.55790986922228	2.54718869262481	3.20775986509782
H	4.23943798812904	1.58415031404083	3.61646111417572
H	0.26105729219245	-0.55291639512235	4.94169456261745
H	0.25763448098695	0.74725855026525	3.74407554477477
H	3.65897114489685	3.15717439974909	3.08585262972242
H	5.20277170494345	3.03259688899069	3.93996509261151
O	4.38934287839006	-1.58894410048204	5.25965299257933
N	3.10003704228784	-3.73646575206861	4.20286654849701
H	2.77383830678348	-4.54984198075379	3.69399610013173
H	2.93444190144763	-2.92355750318881	3.61661401949957
C	5.03436078076971	-2.60140923016738	5.12537954842474
C	4.50426443488610	-3.87208602535815	4.49797048562931
H	4.62336175627021	-4.66450802731491	5.24852196977413
H	6.72196189271075	-1.81243532690081	5.53042929034022
O	6.30712683320273	-2.72130509833865	5.46629563486259
C	5.41282548343409	-4.29857542110034	3.33434740526523
S	5.86121403425911	-3.05459336055093	2.08012988464221
H	4.92552023009817	-5.10907689250109	2.79084257331027
H	6.36653856102887	-4.66843781328814	3.71154142309102

H	6.99804142495134	-2.31014365993471	2.73698970783667
H	8.81539104016980	-2.89796587104123	1.36671699309793
C	9.20135965185984	-2.09484995048916	1.98868383280885
N	8.35380758892576	-1.67495371007670	2.95746224866752
C	10.43787992978841	-1.58532672606237	1.76416097728017
C	8.74450316823416	-0.69233562926130	3.76568672256079
S	7.79643903203357	-0.06379588039350	5.03388541303653
H	11.07545674504870	-1.95828935066904	0.97526891978476
C	10.89977057543127	-0.51664589286516	2.59502946156926
N	9.97682855196565	-0.14981748479238	3.56833693980972
O	11.97702928639055	0.06626384123832	2.52524895416996
H	10.25743489876025	0.59287723700739	4.19569158809690

AuIMesSpym – TS2^{NPT}

Coordinates from ORCA-job

H	5.73204914806756	1.69943563299771	-1.94095939201251
H	4.45140149762027	0.74121405458202	-1.17634171847456
C	5.01217106828099	1.67771799413152	-1.12276776565181
H	7.59313570485261	2.34242169109910	-0.66890060781278
H	2.54239712909715	-2.38321487621153	-0.98894817970478
H	1.01684410286332	-2.02744393390800	-1.80057065668113
C	1.64265649518425	-1.76118361564875	-0.94752961191970
H	4.29557547649829	2.48750847601385	-1.28648606132257
Au	4.39716208493786	-1.71611382976281	1.89907990127911
H	-0.10680533814775	-3.70725684420920	-0.45741656273396
C	7.05330183776208	2.16168080575399	0.25583334327491
C	5.71508514785203	1.81027589374686	0.19273062079183
C	0.03035799454213	-3.08264908691690	0.42005994390914
C	0.90379770747428	-1.99977654881449	0.33377565794971
H	9.37519695186199	3.57389814593253	0.98903264625518
H	-1.80875197587802	-4.94434053602838	0.65315649653046
H	1.96082466550922	-0.72546013391721	-1.06444484050333
H	-1.17529432436968	-5.34822633239930	2.24714351596651
C	-1.60853582715250	-4.54205596265227	1.64679466065091
C	3.26281728829207	-0.10613476499919	1.50511639687702
C	-0.67534421876090	-3.37162715734080	1.57914546366453
C	9.18610996896986	2.62392913216813	1.49577526478910
C	7.72732225092613	2.28323074515786	1.47316623254308
C	1.05215264182739	-1.19860002129661	1.46330275242981
C	5.04484518814272	1.58061530082239	1.40250962182893
H	9.76663715572069	1.85741051302213	0.97498645499752
N	1.91978385702825	-0.06063791890346	1.39364227215273
N	3.67320752863724	1.16889637410291	1.35810241591504
C	-0.49007577144008	-2.54803119340174	2.68621843139627
C	0.36883822890859	-1.45800978899076	2.65607093996476

C	7.01756052739690	2.07323727024333	2.64326443687472
C	5.66519209343063	1.72634863965405	2.63704816272677
H	-2.55655975615371	-4.26547087057624	2.11313769200514
C	2.60069759696274	2.00325111330719	1.14660229974027
H	9.55995199777303	2.69846462888590	2.51756310081500
C	1.49415262094036	1.22916100557678	1.17530095280314
H	-1.02228399130891	-2.76578290791416	3.60771809189699
H	1.26003695859390	0.18289088048624	3.75551385750026
C	0.54762989668242	-0.62910226923823	3.89370275942083
H	7.51810566301130	2.17982912053378	3.60078959268571
H	0.45220218647396	1.47949489826809	1.06814252316419
H	2.71107725669176	3.06600282869399	1.01153812326537
C	4.94689304842678	1.51796666034069	3.93283257791391
H	5.41153415622904	2.11053029235984	4.72108441042226
H	0.89765677855337	-1.26090133381012	4.71366921355230
H	-0.40213705841008	-0.18743961766620	4.20546298634198
H	4.99913710451299	0.47029153167885	4.23975613721561
H	3.89076405444862	1.78542630520763	3.87472022536845
O	6.57305513515289	-1.06068800938776	5.39907982969836
N	4.14657933201600	-2.34459003138660	5.22882127711738
H	3.33558431130604	-2.94580737797646	5.30833122655426
H	4.06086406916975	-1.81951229413587	4.36371411375406
C	6.58789262912298	-2.26893349373446	5.41487993710756
C	5.34781900614935	-3.13402110248896	5.24560772164400
H	5.31728937315779	-3.77313751972360	6.13762206935708
H	8.53377336295386	-2.50579646560735	5.42095933567650
O	7.66726303487271	-3.01483579468068	5.57285858464050
C	5.50965088851589	-4.13912129882203	4.09914706884917
S	5.74135445999995	-3.54491263399566	2.39330135572425
H	4.61140552116567	-4.75427103797723	4.01903114891825
H	6.36003390361351	-4.79844493437392	4.27520558939389
H	6.91761619036086	-2.87162115630794	2.47324823849475

S	10.28592891576583	-1.65506264918464	4.74976705817527
C	10.01390598525409	-1.55564218745968	3.04786846733991
N	11.07251315934027	-1.20274698474420	2.27741236409265
N	8.79359105194153	-1.80893083570053	2.54057101191481
C	10.89164633926913	-1.14685209446154	0.97055393684268
C	8.63276657708003	-1.69300807196869	1.22355201297594
C	9.66626787956210	-1.38797233911393	0.36318882298266
H	11.76060404056235	-0.88583597022856	0.37009479583047
H	7.62572833144254	-1.83404819061360	0.83604880818102
H	9.53122170285345	-1.31729791608565	-0.70750955533178

AuIMesSbtz – TS2^{NPT}

Coordinates from ORCA-job

H	4.39522295968869	1.05468207840344	-3.04453139022122
H	3.83293180858261	0.06447450441395	-1.69009406833602
C	4.01076803102330	1.09005061907105	-2.02538111126031
H	6.41289602744466	2.13674043793833	-2.67072702749377
H	1.67814507722795	-1.63179871921098	-0.50755219476901
H	0.08137979215072	-2.38951425154949	-0.59995059761532
C	0.65583548881692	-1.59668917316549	-0.12127101630386
H	3.04109463550680	1.59470493176147	-2.04215901870172
Au	4.63531221204916	-1.19089718282687	1.31203690779769
H	-0.56971235843451	-3.52503729544144	1.31569962318997
C	6.18549780854204	2.27829077548131	-1.61858700776832
C	4.97883631298346	1.79443082331369	-1.12612204562143
C	-0.05204149107352	-2.81785005385428	1.95642604427982
C	0.63292199981027	-1.76077871020905	1.36689149584376
H	8.31452739501730	3.69952575466420	-2.40693694324738
H	-0.97739218887869	-4.94715558016961	3.24269037255264
H	0.23141694712550	-0.63592442625588	-0.42463263200862
H	-0.36209085983521	-4.49777422381299	4.84461912461407
C	-0.85740204696669	-4.12263199339174	3.94711828118354
C	3.26214311416010	0.27337449400599	1.24245031137621
C	-0.08791180742187	-2.99192540357943	3.33547835472692
C	8.40577851187999	3.42403042395334	-1.35483775644260
C	7.09941980016766	2.93907514143317	-0.80426291463535
C	1.29566975749067	-0.86912708778368	2.20930705174604
C	4.70681981251950	1.99076372582978	0.22771652802237
H	9.16646138923447	2.63980456921097	-1.28723279904045
N	1.97241136592780	0.25253572667492	1.63025039526305
N	3.46434126279784	1.51505654061918	0.75860361340438
C	0.59333536596219	-2.08143264846259	4.13699324697775
C	1.29215100497896	-1.00790125446562	3.59746512666853

C	6.78121913160005	3.12193674620073	0.53729888381518
C	5.58832835324702	2.65637847300100	1.07894825508560
H	-1.85849390630891	-3.79232116417792	4.24187174959068
C	2.31251671410773	2.26618544262076	0.84213900521693
H	8.77441559507904	4.28627515422717	-0.79687640467621
C	1.37175791240730	1.47028135216686	1.39128187202111
H	0.58449020748839	-2.20811270779672	5.21523237460250
H	3.08505904852870	-0.01681564928157	4.25106620659430
C	2.01638898550670	-0.04040846804176	4.48146671920617
H	7.47869012594736	3.64448637638959	1.18471873054560
H	0.33813833480831	1.65263877534497	1.63264596731170
H	2.27073949179380	3.28828658980614	0.50532060587257
C	5.26887253614051	2.86667038805633	2.52661516342566
H	5.12524262781398	1.91374533375328	3.04332778810380
H	1.89770745262325	-0.31913858470797	5.52831537097381
H	1.63869557599343	0.97760658753055	4.35376134630804
H	4.34884597522642	3.44321112051212	2.65417320598421
H	6.07843474121447	3.40377256642089	3.02046746165333
O	4.84681127794805	-3.17754298681085	4.62011306100087
N	3.50072808315444	-4.22341090918004	2.48082909263139
H	3.20704816456388	-4.50127340525058	1.55136361687176
H	3.44783592379213	-3.20954810871206	2.52403946628701
C	5.33256939999114	-4.12058414738821	4.04836495782181
C	4.84743144198876	-4.67282647740234	2.72741795710668
H	4.83242465296037	-5.76377244450816	2.83800616740284
H	6.69245142939209	-4.37335475599353	5.32398442213661
O	6.38337104285990	-4.79661657674683	4.50753040544345
C	5.87648534467428	-4.42388683762057	1.61305523630210
S	6.36682149124533	-2.71770506197141	1.21747756403002
H	5.48023227853982	-4.84608793021099	0.68850610870296
H	6.81602024610711	-4.93171599570275	1.84246081669772
H	7.29272386429278	-2.41773546376166	2.37263516436253

H	6.39011280507161	-0.27986625769235	3.88308200379820
C	7.25500441471584	-0.24326048059509	4.53622969189499
H	6.55755847559794	1.42608128594613	5.67275220575385
C	7.35706190674695	0.70599350423523	5.53919464833994
N	8.33824666732797	-2.16254220058781	3.42848422671029
C	8.28871380557247	-1.16411572291860	4.37280063763627
C	8.47042269319098	0.75047262374709	6.37957516767479
C	9.43358991646257	-2.89395338075480	3.48049042071921
S	9.85196675875940	-4.19619133200438	2.48909004444865
C	9.40776076746553	-1.10899910766348	5.22279363599115
H	8.52805605476095	1.50206109185836	7.15842687041183
C	9.50759625202406	-0.15782873870760	6.22805815190578
S	10.51196899878729	-2.36475842854339	4.76987767407333
H	10.37400981030613	-0.12451262967889	6.87827832802533

AuIMesStzn – TS2^{NPT}

Coordinates from ORCA-job

H	4.91448826638432	-0.11814028982348	-0.95620103672910
H	4.36593093929624	1.42720827415051	-1.59119551468368
C	5.21240552025674	0.92138296615401	-1.11906720473820
H	7.59571895671847	1.99982674653480	-0.46133674421597
H	1.14475224481909	-2.74283775510503	-1.80931188227406
H	1.09003865399699	-0.99201379100550	-1.56588109129509
C	1.38787675202127	-1.94104994505609	-1.11233802335454
H	6.04881994707017	0.93169458461181	-1.81770854051338
Au	4.15153097506948	-1.53592180853938	2.07813602674283
H	-0.24155830872586	-3.97706718975400	-0.41938949426649
C	6.87957966883984	2.08935689125550	0.35000016681191
C	5.59972233187094	1.58218954248702	0.16755271992340
C	-0.11600048017388	-3.27177514790379	0.39628563125600
C	0.69786973824263	-2.16184222531693	0.19823189926508
H	9.39112278341870	2.43306269409806	1.61649148752568
H	-1.37515158227146	-5.50309138180506	1.11581129673088
H	2.47405022422658	-1.91108188877938	-0.98912862499903
H	-1.64237561043677	-5.04994386813375	2.80992651585710
C	-1.66285760225301	-4.68714594559480	1.78070096283552
C	2.99056876378932	-0.06122721893472	1.36878216130121
C	-0.76597477214897	-3.49998939487735	1.60440246174705
C	8.65502320867444	3.23724424002216	1.70383642523415
C	7.26402026942365	2.70572554813220	1.53815647272393
C	0.85092665822782	-1.27344904579996	1.26195411282336
C	4.69684742044412	1.70533904767374	1.22524667116115
H	8.78361111929057	3.71279456966586	2.67675493682627
N	1.67625778983198	-0.11661017812063	1.08229370216676
N	3.36839614087657	1.19431799977664	1.06279301691409
C	-0.58284730457243	-2.58751601224519	2.63857161733117
C	0.22065250470468	-1.46285375610698	2.49180718022314

C	6.33011861880372	2.81187802431099	2.56128110472220
C	5.03536756098262	2.31825305253317	2.42959817617083
H	-2.69959198751754	-4.42366921395835	1.54840125935998
C	2.29968529442561	1.92136325958085	0.58565908113597
H	8.88857525662328	3.97171440816293	0.92848240693539
C	1.23243069358703	1.09495690455196	0.59866138532766
H	-1.07769731703065	-2.75348389421956	3.59057044924801
H	1.45007958753219	-0.46366937350191	3.94542893781346
C	0.40545402519156	-0.49994536113583	3.62359357483498
H	6.61249338449023	3.29143603750060	3.49330908064974
H	0.20657090188445	1.25473319371706	0.31220772021477
H	2.39698463390043	2.95110128321398	0.28544724175287
C	4.05241838858074	2.43757284335222	3.55286130514848
H	3.76231901127166	1.45245159467273	3.92966628555214
H	-0.20381387422854	-0.79568097670784	4.47743176339448
H	0.12371700530189	0.51637962663273	3.33653239685165
H	3.13663941152766	2.94161089814109	3.23415194907840
H	4.48493789599863	3.00281377956071	4.37819478137142
O	6.25654183181851	-0.49068688587987	5.08304544908054
N	3.92548377245369	-1.82602570976688	5.52043708438602
H	3.12726428916639	-2.44981686245896	5.49309403759314
H	3.87094672700671	-1.22219218044171	4.70541703817385
C	6.34310874877754	-1.66437180597808	5.36231139742054
C	5.14981075610302	-2.58460407437301	5.51163712999264
H	5.25531814913809	-3.08524597070528	6.48222397000808
H	8.25477427989262	-1.78747651995329	5.13410459876309
O	7.47816456402776	-2.31595790900934	5.51872852169608
C	5.26032265237837	-3.72019285773536	4.48137891889880
S	5.52436672542387	-3.27679674345697	2.73619627364842
H	4.34041117915794	-4.30648804079895	4.49254130224917
H	6.08981391729720	-4.38108860135604	4.73340396077784
H	6.83394749264353	-2.64725727215931	2.72975838965758

S	10.39407000177416	-0.92459644645558	1.19123638521745
C	9.27369863856456	-1.54968000048091	-0.08166805608264
H	8.98074690601397	-0.73180263976579	-0.73880832036894
H	9.78556094065916	-2.30726359637137	-0.67357878507045
C	8.06698622908337	-2.13542791750210	0.68095744220488
H	7.14469909139603	-1.62720879687963	0.37959199884272
H	7.93966856382557	-3.19373858437612	0.43244019542257
N	8.22489355403800	-2.00545136962860	2.11848562602836
C	9.30871658994473	-1.43264272738981	2.50172973484079
S	9.85120769117473	-1.09068083514425	4.09201349872454

REFERENCES

(1) Reich, H. *pKa Values in DMSO Compilation*.

(2) Oliveira, I. S.; Garcia, M. S. A.; Cassani, N. M.; Oliveira, A. L. C.; Freitas, L. C. F.; Bertolini, V. K. S.; Castro, J.; Clauss, G.; Honorato, J.; Gadelha, F. R.; Miguel, D. C.; Jardim, A. C. G.; Abbehausen, C. Exploring Antiviral and Antiparasitic Activity of Gold N-Heterocyclic Carbenes with Thiolate Ligands. Dalton Transactions 2024, 54, 18963-18973. https://doi.org/10.1039/d4dt01879f.

(3) De Frémont, P.; Scott, N. M.; Stevens, E. D.; Nolan, S. P. Synthesis and Structural Characterization of N-Heterocyclic Carbene Gold(I) Complexes. Organometallics 2005, 24 (10), 2411–2418. https://doi.org/10.1021/OM050111C.
